# Decomposing bulk signals to reveal hidden information in processive enzyme reactions: A case study in mRNA translation

**DOI:** 10.1371/journal.pcbi.1011918

**Published:** 2024-03-05

**Authors:** Nadin Haase, Wolf Holtkamp, Simon Christ, Dag Heinemann, Marina V. Rodnina, Sophia Rudorf

**Affiliations:** 1 Leibniz University Hannover, Institute of Cell Biology and Biophysics, Hannover, Germany; 2 Max Planck Institute for Multidisciplinary Sciences, Department of Physical Biochemistry, Göttingen, Germany; 3 Paul-Ehrlich-Institut, Division of Allergology, Langen, Germany; 4 Leibniz University Hannover, Hannover Centre for Optical Technologies (HOT), Hannover, Germany; 5 Leibniz University Hannover, Institute of Horticultural Production Systems, Hannover, Germany; 6 Leibniz University Hannover, PhoenixD Cluster of Excellence, Hannover, Germany; Georg-August-Universitat Gottingen, GERMANY

## Abstract

Processive enzymes like polymerases or ribosomes are often studied in bulk experiments by monitoring time-dependent signals, such as fluorescence time traces. However, due to biomolecular process stochasticity, ensemble signals may lack the distinct features of single-molecule signals. Here, we demonstrate that, under certain conditions, bulk signals from processive reactions can be decomposed to unveil hidden information about individual reaction steps. Using mRNA translation as a case study, we show that decomposing a noisy ensemble signal generated by the translation of mRNAs with more than a few codons is an ill-posed problem, addressable through Tikhonov regularization. We apply our method to the fluorescence signatures of *in-vitro* translated LepB mRNA and determine codon-position dependent translation rates and corresponding state-specific fluorescence intensities. We find a significant change in fluorescence intensity after the fourth and the fifth peptide bond formation, and show that both codon position and encoded amino acid have an effect on the elongation rate. This demonstrates that our approach enhances the information content extracted from bulk experiments, thereby expanding the range of these time- and cost-efficient methods.

## Author summary

Ensemble (bulk) *in-vitro* experiments are less complex and expensive than single-molecule measurements, but their read-outs are blurred due to the stochastic nature of biomolecular processes. Our study investigates how information about individual steps in processive reactions can be deduced from ensemble experiments, despite these inherent challenges. We introduce a least-squares method for decomposing ensemble signals from synchronized enzymatic reactions, allowing us to reveal distinct features of individual reaction steps. Using mRNA translation as a showcase, we propose a systematic approach to assess the feasibility of ensemble signal decomposition. To demonstrate the efficacy and limits of our approach, we decompose simulated fluorescence signatures of *in-vitro* mRNA translation processes. We then apply our method to experimental data of LepB mRNA translation, where a codon-by-codon analysis reveals that both the codon position and the encoded amino acid impact the translation dynamics. Our approach can be applied to assess bulk signals of other processive enzymatic reactions beyond mRNA translation, provided a comprehensive quantitative understanding of the process is available. The introduced method offers a robust means to extract detailed information from bulk experimental setups, thus helping to bridge the gap between cost-effective bulk experiments and detailed single-molecule studies.

## Introduction

**Fig 1:**
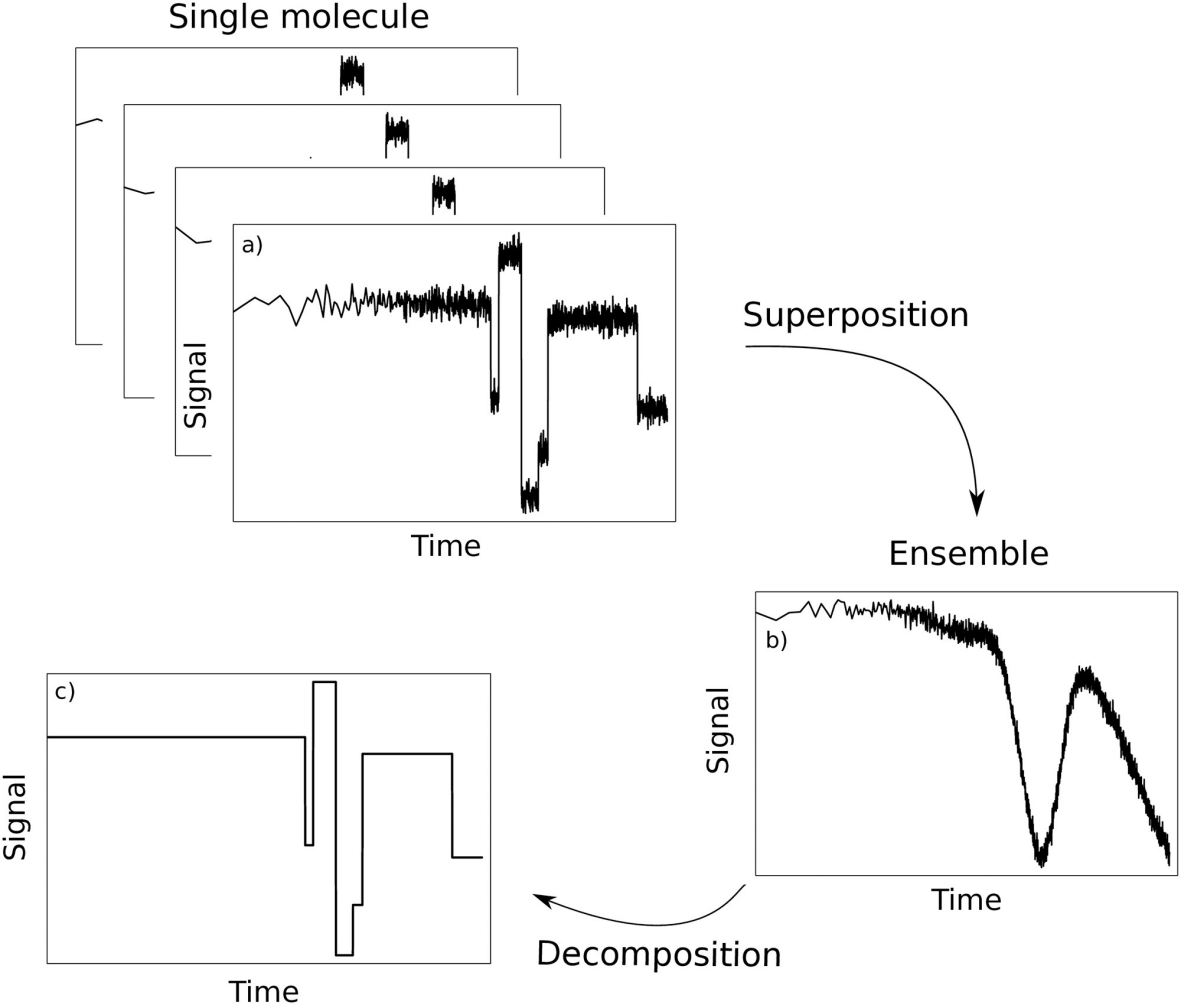
Schematic representation of the decomposition of ensemble time traces. a): The action of an individual processive enzyme is monitored over time. Changes in the signal are interpreted to obtain a detailed picture of step-by-step transitions and related kinetics of this molecule. b): Ensemble time trace for a large number of enzymes in parallel. The detected signal is a superposition of all individual signals. Due to the stochasticity of biochemical processes, these individual signals differ from each other to some extend. Consequently, the features of the individual signals are blurred or even lost during superposition. c): The ensemble time trace needs to be computationally decomposed to reveal the hidden information on the transitions of a single molecule.

Many biochemical reactions are driven by processive enzymes that catalyze multiple rounds of reactions while staying attached to the same polymeric substrate molecule. Prominent examples of processive enzymes are DNA and RNA polymerases, ribosomes, cellulases, or endonucleases [1, 2]. To study the kinetics of the biochemical processes driven by processive enzymes, basically two distinct approaches can be taken: single-molecule experiments and ensemble studies [3–5]. In single-molecule experiments, the action of an individual enzyme is monitored over time, which results in a detailed picture of the step-by-step motion of this molecule (see Fig 1). Because thermal fluctuations cause differences in the molecular activities, single-molecule experiments have to be performed on a multitude of individual enzymes to obtain a complete picture of the biochemical process. This need for repetition in combination with an often complicated setup can make single-molecule experiments a technical and financial challenge. In comparison, ensemble (bulk) experiments are relatively cost- and time-efficient. Their results have a high statistical power, because they are performed on a large number of enzymes in parallel. However, the signal of an ensemble experiment is typically a superposition of all individual signals, each of which is emitted by a single enzyme in the reaction volume. Due to the stochasticity of processive biochemical processes, these individual signals differ from each other to some extend. Consequently, features or characteristics of the individual signals are blurred or even lost during superposition and, thus, are not present in the ensemble signal [6, 7]. The more time has passed after start of the experiment, the more pronounced is this blurring, even for initially synchronized ensemble reactions. Therefore, ensemble time traces need to be computationally decomposed to reveal hidden information about the underlying enzymatic process [8]. A meaningful computational analysis of ensemble data relies on a mathematical model that fulfills two requirements: First, the model has to reproduce the experimental data and, second, it must do so with an appropriately limited set of parameters. In general, an under-parameterized model lacks the complexity required to reflect the true biochemical process, whereas model over-parameterization leads to overfitting, which causes ambiguous and unreliable results [9, 10]. However, which number of parameters is appropriate is highly case-specific.

In the context of molecular biology and biochemistry, fluorophores are used for different purposes, e.g., for visualization of cell compartments or localization of compounds [11, 12]. Because the intensity of the light emitted by a fluorophore depends on the physico-chemical properties of environment *via* quenching and de-quenching, fluorophores can also be applied as molecular probes for state transitions in biochemical reactions [13–18]. For the latter case, it is required that to each state of the reaction process a specific fluorescence intensity can be assigned. We refer to these state-specific fluorescence intensities as “intrinsic fluorescence intensities” (IFIs). The aim of the decomposition of an ensemble fluorescence time trace is to determine these state-specific signal intensities for the individual states. For the sake of clarity, we restrict our analysis to processes that can be assumed to have a fixed (non-stochastic) number of rounds of reactions, i.e., where fluctuations in the processivity can be neglected (e.g., template-directed reactions such as mRNA translation or nucleic acid polymerization). Furthermore, we assume that the state-specific signal intensities are the only parameters to be determined by the decomposition procedure and that the kinetic rates related to the action of the enzyme do not need to be inferred from the particular ensemble time trace that is to be decomposed. Of course, determination of IFIs by signal decomposition can be part of an extended experimental and model fitting strategy that in addition also allows for the fitting of kinetic parameters, as we have shown [8].

A well-known example for a processive biochemical process is messenger RNA (mRNA) translation, i.e., the biosynthesis of peptides by molecular machines called ribosomes [19]. Briefly, during translation initiation a ribosome binds to mRNA, i.e., single-stranded RNA which consists of a sequence of trinucleotides called codons that encode for the different amino acids. After binding to the mRNA, the ribosome moves from codon to codon catalyzing the peptide bond formation of the corresponding amino acid molecule and the nascent peptide chain. Translation of the mRNA is finished when the ribosome reaches the stop codon and gets released from the mRNA. Premature drop-off of ribosomes from mRNAs is a rare event (probability of roughly 10^−4^ per codon [20]), therefore the processivity of a translating ribosome is mostly simply determined by the length of the mRNA, i.e., the number of codons the mRNA consists of. Except for a few specific cases [21–26], directly assessing the kinetics of mRNA translation in its natural *in-vivo* environment, i.e., in the living cell, is currently infeasible without heavily disturbing the system. However, the movement of ribosomes along mRNAs can be indirectly monitored in *in-vitro* translation experiments by using fluorescent molecules that are attached to the N-termini of the nascent peptides, see ref. [8] for details. The experiments are constructed in a way such that each mRNA is translated only a single time: Once a ribosome reaches the end of the truncated mRNA, it is bound to remain there and does not engage in another round of translation. This yields a unique fluorescence time trace for each translated mRNA that we refer to by the mRNA’s “fluorescence signature”. All ribosomes are initially synchronized and are simultaneosly translating identical mRNA sequences [8]. The ensemble fluorescence time trace (or ensemble fluorescence signature) is a superposition of all fluorescence signals. Translation is a stochastic process, thus the ensemble fluorescence signature obtained from a bulk experiment is much smoother than the individual fluorescence time traces generated by single mRNA translation processes, which consist of discrete gradations [8]. The goal is to find the fluorescence intensities that are associated with the individual states of the translation process from the ensemble fluorescence signature.

In this work, we investigate the feasibility of the decomposition of ensemble time traces that result from the simultaneous action of a multitude of identical, initially synchronized ribosomes. In particular, we consider the typical processivity of the enzymes and analyze its impact on the meaningfulness and truthfulness of ensemble signal decomposition. At first, we show that time traces of low-processivity reactions can be decomposed with good fidelity, in contrast to reactions with higher processivity. We then analyze this phenomenon in terms of the condition number associated to the underlying biomolecular process. Finally, we show that the application of a regularization method can expand the applicability of ensemble signal decomposition to reactions with higher processivity.

## Results

### Decomposition of time traces from low-processivity reactions: Fluorescence signatures of mRNAs consisting of only four codons can be fitted by standard least-squares approach

At first, we study the consecutive translation of only four codons by the ribosome, which can be seen as an enzymatic action with comparably low processivity. To assess the quality of the decomposition results and to precisely quantify the deviations of output (fitted) from input (assigned) parameter values, we use simulated ensemble fluorescence signatures instead of experimentally obtained data: We first generate artificial ensemble fluorescence signatures from mRNAs that consist of four identical codons. Then, we “forget” all IFI values that were used to generate the data and, instead, apply fluorescence signature decomposition to obtain estimates for the IFIs.

For data generation, we simulate translation as a continuous-time Markov process where the states comprising the Markov chain represent the steps that a ribosome undergoes during translation. Each state in the Markov process is associated with a respective IFI. The Markov model used in this work includes 6*n* + 2 states and *n* + 3 assigned IFIs, where *n* is the number of codons of each mRNA. Therefore, the Markov model for translation of an mRNA consisting of four codons includes 26 states and seven IFIs. The IFIs are assigned to the states in a way such that they represent expected changes in fluorescence intensity during the biochemical process of translation, e.g., after translocation of the ribosome to the next codon. Therefore and in the interest of fit parameter reduction some states are assigned the same IFI value. For further information on the Markov model and the assignment of the IFIs, see [8] and Methods. Using our Markov model of translation, we calculate the time-dependent state occupancy probabilities **P**, see Methods. These probabilities express how likely it is to find a ribosome at a specific time in a certain state. Furthermore, we construct IFI input vectors ${\vec{x}}_{\text{in}}$ consisting of *n* + 3 random positive real numbers. The simulated input ensemble fluorescence signature ${\vec{s}}_{\text{in}}\left(t\right)$ is then given by the sum over all components of the IFI vector ${\vec{x}}_{\text{in}}$ weighted by the time-dependent state occupancy probabilities **P** of the Markov process:

$$\begin{array}{c} {\vec{s}}_{\text{in}}\left(t\right)≔P\;{\vec{x}}_{\text{in}}. \end{array}$$ (1)

To imitate experiments in which thermal fluctuations modulate the fluorescence signal, we overlay the simulated input fluorescence signature ${\vec{s}}_{\text{in}}\left(t\right)$ by a Gaussian random noise $\vec{\Sigma }$ such that resulting final simulated data are given by $\vec{s}\left(t\right)≔{\vec{s}}_{\text{in}}\left(t\right)+\vec{\Sigma }$, see Methods.

**Fig 2:**
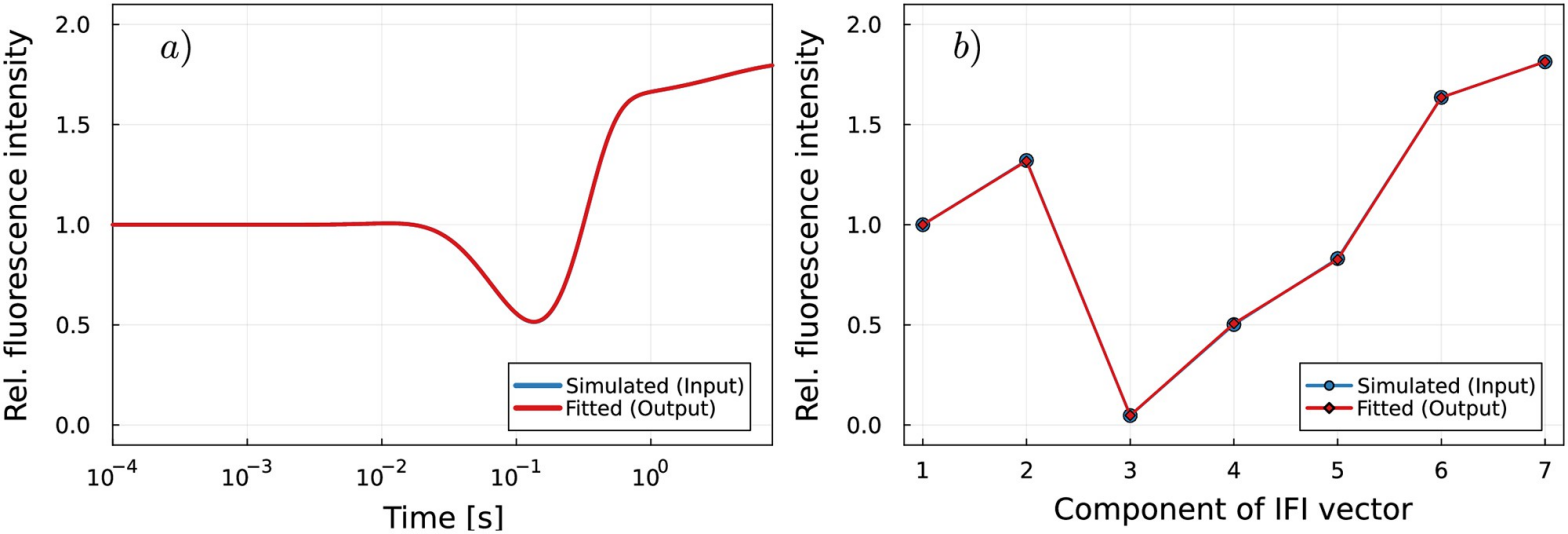
Fluorescence signature of an mRNA that consists of four identical codons for random IFI input vector. a): The simulated fluorescence signature is compared to the best theoretical fit in terms of least squares. The theoretical model and simulated data curves are in perfect agreement. b): Fitted IFIs obtained from the analysis of the fluorescence signature compared to the given IFI input vector.

To test our decomposition strategy for the simulated ensemble fluorescence signature, we pretend that all state-specific IFIs are unknown. Analogously to experimental data analysis, where short-term fluctuations are smoothed out, the simulated data $\vec{s}\left(t\right)$ are also smoothed by a moving average operation, i.e., ${\vec{s}}_{\text{sm}}\left(t\right)≔\text{smooth}\left(\vec{s}\left(t\right)\right)$. Fig 2a) shows an example for a smoothed simulated ensemble fluorescence signature ${\vec{s}}_{\text{sm}}\left(t\right)$. The smoothing step is necessary to fit the model using a least-squares solver to obtain the output IFI vector ${\vec{x}}_{\text{out}}$

$$\begin{array}{c} P\;{\vec{x}}_{\text{out}}={\vec{s}}_{\text{sm}}\left(t\right), \end{array}$$ (2)

see Methods for details.

Ideally, a perfect fit would reveal the IFI values that were used to generate the fluorescence time traces, i.e., ${\vec{x}}_{\text{out}}={\vec{x}}_{\text{in}}$. However, due to the added noise, the output and input IFI values ${\vec{x}}_{\text{out}}$ and ${\vec{x}}_{\text{in}}$ may slightly deviate. Figure A in S1 Text shows that the uncertainty is lower in fitted IFI values that correspond to the states in the beginning and towards the end of the studied process. This inhomogeneity in the fit quality is a consequence of the constraints given by the particular “imitated” experimental setup [8]: First, the translation processes are initially synchronized in the sense that all ribosomes simultaneously start to translate the mRNA molecule that they are bound to. By definition, the intrinsic fluorescence intensity that corresponds to the initial state is set to IFI_1_ = 1, i.e., it serves as a reference for the normalization of all fluorescence values. Second, all ribosomes end up and remain in the same last “end” state. Therefore, the IFI corresponding to the “end” state can be well determined as long as the process is monitored long enough, see also Fig B in S1 Text.

In summary, time traces from initially synchronized biochemical reactions with low processivity can be decomposed by standard least-square approaches, and even subtle differences in intrinsic fluorescence intensities of different states can be detected, see Fig 2b).

### Standard least-squares approach is not sufficient to decompose time traces from higher-processivity reactions

**Fig 3:**
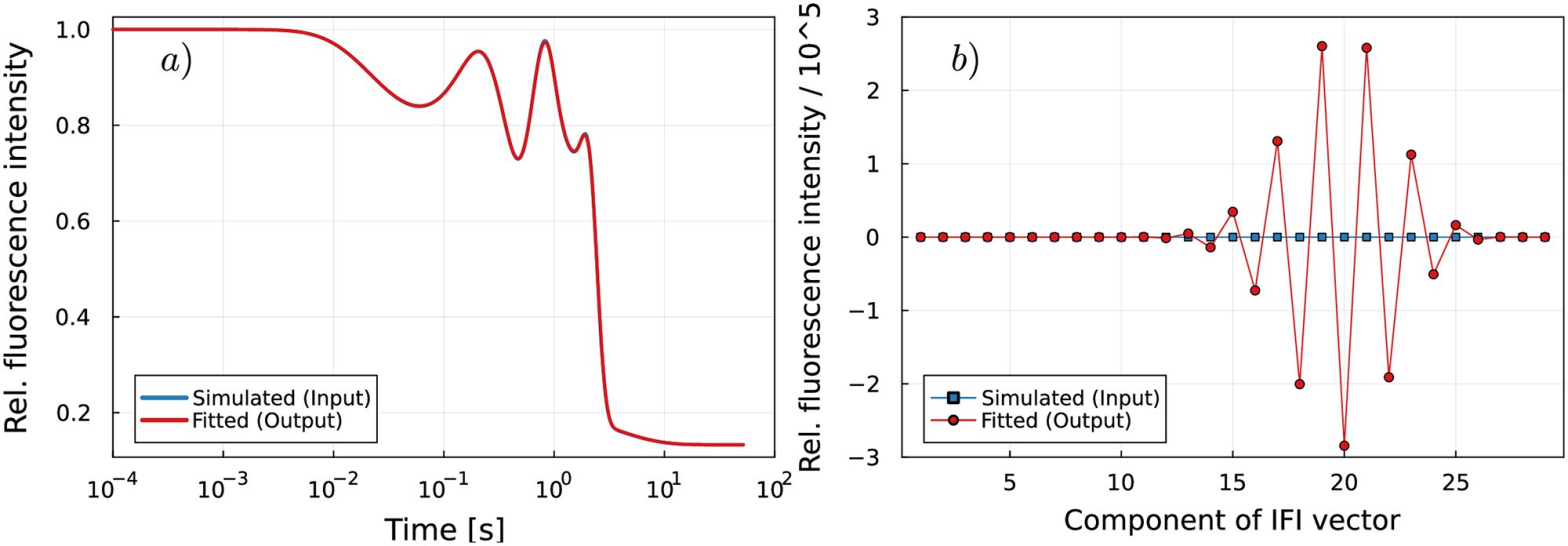
Fitting fluorescence signatures of longer mRNAs reveals signs of an ill-posed problem. a): Fluorescence signature of an mRNA that consists of 26 identical codons for a random IFI input vector. The simulated fluorescence signature is compared to the best theoretical fit in terms of least squares. No regularization is applied. b): Fitted IFIs obtained from the decomposition of the fluorescence signature in a) compared to the given IFI input vectors. No regularization is applied.

**Fig 4:**
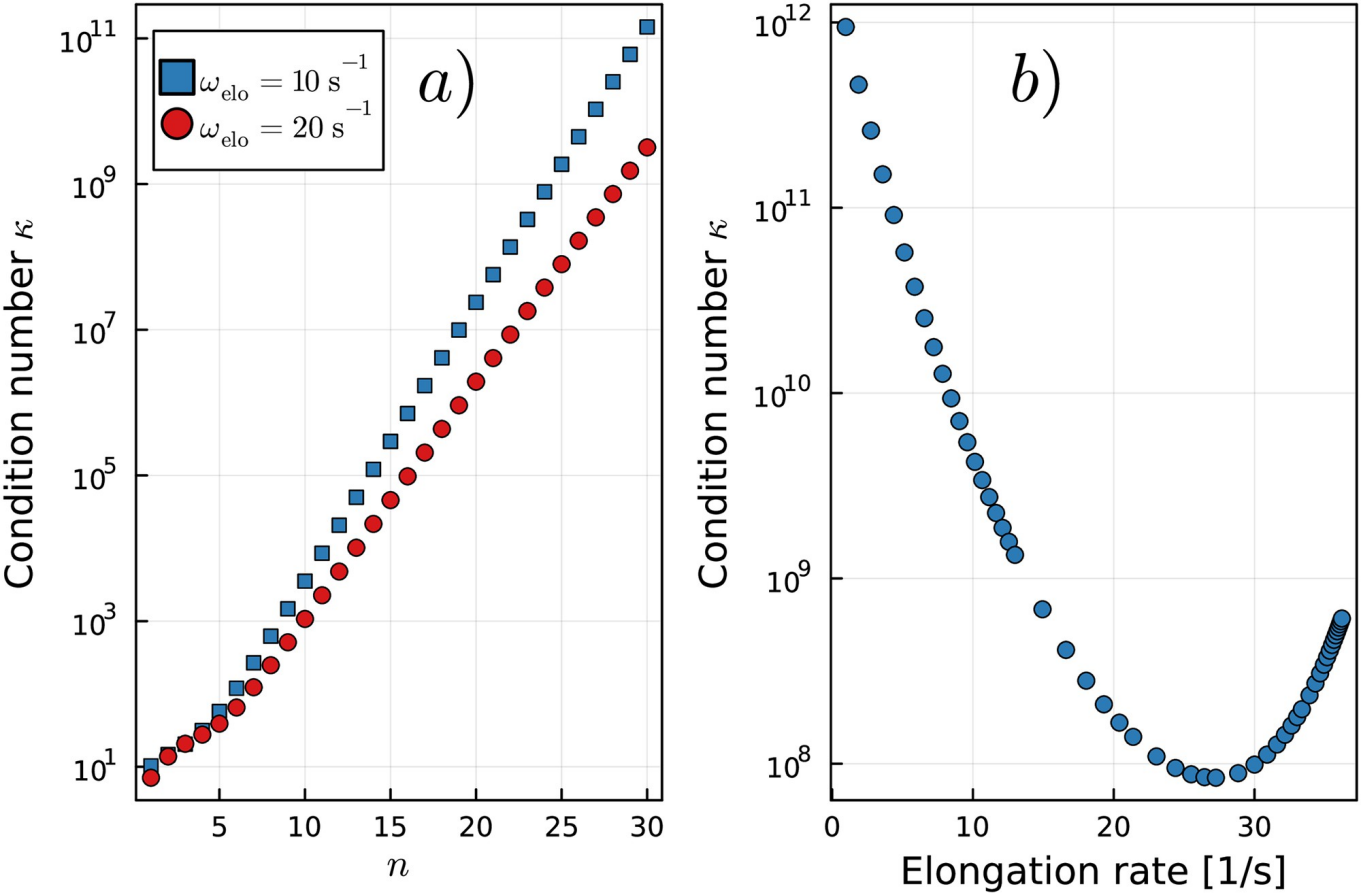
Characteristics of the occupancy probabilities matrix P. a): Condition number *κ* for translation of mRNAs consisting of *n* identical codons with elongation rates *ω*_elo_ = 10*s*^−^1 and *ω*_elo_ = 20*s*^−^1. b): Condition number *κ* for translation of an mRNA that consists of 26 identical codons for various elongation rates *ω*_elo_.

In this paragraph, we show that signal time traces from reactions with higher processivity generally cannot be decomposed by standard least-square methods and that, instead, more elaborate data analysis methods are required to obtain meaningful fits. To show the limits of the standard least-square approach, we proceed as described in the previous paragraph. However, here we analyze simulated ensemble fluorescence signatures corresponding to translation of mRNAs consisting of 26 identical codons, see Fig 3a), instead of just four codons as in the previous paragraph. The Markov model includes 6*n* + 2 = 158 states and *n* + 3 = 29 assigned IFIs, with *n* = 26 the number of codons on the mRNA. Decomposition of these ensemble fluorescence signatures by a standard least-square approach reveals IFI values that greatly deviate from the randomly chosen input IFI values that were used to generate the fluorescence data, see Fig 3b). The components of the fitted IFI vector exhibit strong oscillations, span several orders of magnitude, and have negative values. These unreasonable fitting results indicate that the present least-squares problem is ill-posed [27]. To validate this assumption, the singular values *σ*_*i*_ and the condition number *κ*(**P**) of matrix **P** are calculated, see S2 Text for definitions and further details: The singular value decomposition of matrix **P** reveals that the singular values *σ*_*i*_ decay gradually to zero, see Fig C in S1 Text. Furthermore, the condition number *κ*(**P**) of matrix **P** is very large, with a value of *κ*(**P**) = 4.8 × 10^9^. Both criteria imply that the matrix **P** is ill-conditioned, which means that the computed solution of the ill-posed problem Eq (2) is potentially very sensitive to perturbations in the input data, i.e., the presence of thermal noise in the simulated ensemble fluorescence signature $\vec{s}$ [27, 28]. Fig 4a) shows that the condition number grows exponentially with the level of processivity, i.e., the number of repeats of the enzymatic action performed by the processive enzyme. This exponential growth is accelerated when the number of repeats is larger than five. The condition number corresponding to translation of mRNAs consisting of 26 codons is several orders of magnitude higher than the condition number corresponding to translation of four-codons mRNAs. Therefore, solving Eq (2) for the unknown IFI values in the sense of least-squares is feasible for signals from the translation of 4-codon-mRNAs, but is an ill-posed problem for longer mRNAs, e.g., in the case of mRNAs with 26 codons. In addition, the condition number depends on the kinetics of the biochemical reaction, see Fig 4b). Here, the kinetics are determined by the elongation rate of the translating ribosomes, which is reflected in the state occupancy probabilities matrix **P**.

### Application of Tikhonov regularization to restore decomposability of signals from high-processivity processes

To find a meaningful solution of a linear ill-posed problem by the method of least squares, the inclusion of additional information is required, which is commonly done by applying Tikhonov regularization [29]. The purpose of regularization is to include side constraints and provide robust methods for choosing the weight given to these side constraints to solve the ill-posed problem, see S2 Text for an introduction to the method. Briefly, a damping is added to filter out the components corresponding to the small singular values *σ*_*i*_ of the matrix **P** [28]. The amount of applied filtering is controlled by the regularization parameter *α*. For larger values of the regularization parameter *α*, stronger regularization is applied, i.e., more filtering is introduced, the fitted solution vector ${\vec{x}}_{\text{out},\alpha }$ gets smoother and changes only little with higher *α*. Simultaneously, the regularization error increases, which means that some physically meaningful components of the unknown solution are filtered out (regularization error). For a small regularization parameter *α*, the fitted solution vector ${\vec{x}}_{\text{out},\alpha }$ is dominated by terms corresponding to the smallest singular values *σ*_*i*_ and its entries show oscillatory sign changes (perturbation error). In addition, small changes in *α* result in strong changes in the solution ${\vec{x}}_{\text{out},\alpha }$. To choose an optimal value for the regularization parameter *α*, the norm of the regularized solution $\left|\right|{\vec{x}}_{\text{out},\alpha }{\left|\right|}_{2}$ and the residual norm $||P{\vec{x}}_{\text{out},\alpha }-{\vec{s}}_{\text{sm}}{\left|\right|}_{2}$ are plotted against each other. For discrete ill-posed problems, the plot in log-log scale shows a characteristic L-shaped curve with the optimal regularization parameter *α* corresponding to the corner of the curve [27, 30]. The optimal regularization parameter *α* balances the regularization and the perturbation error to find a stable solution.

**Fig 5:**
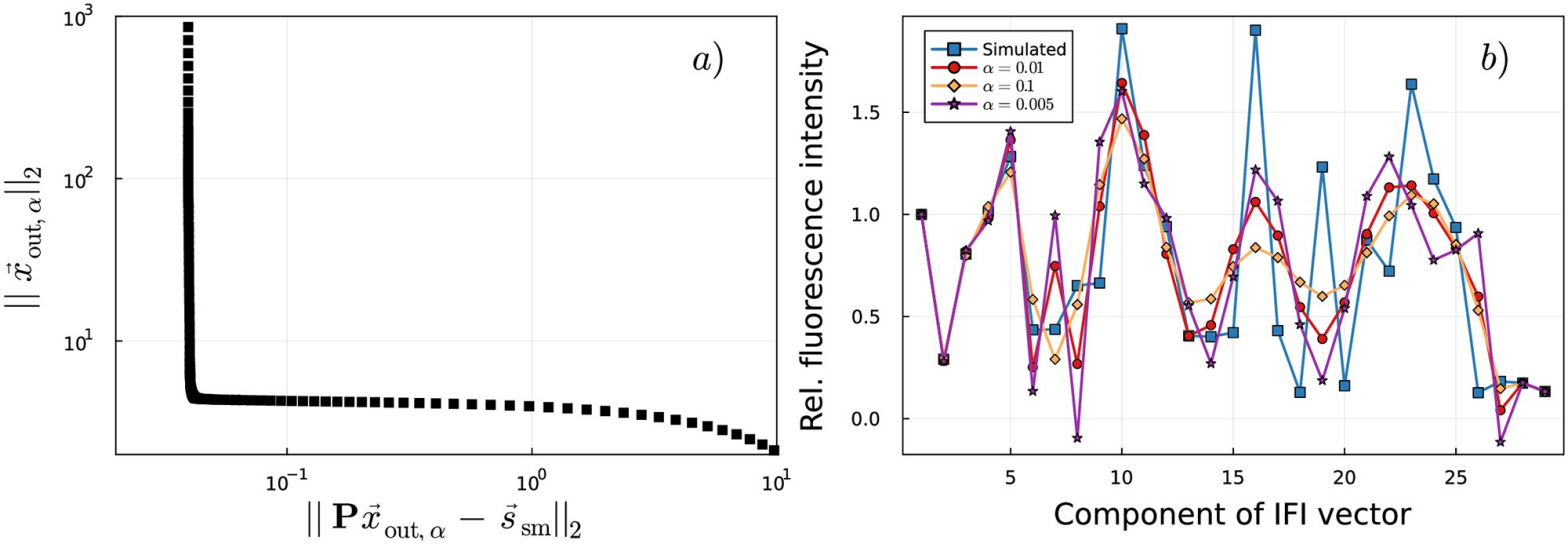
Decomposition of fluorescence signatures of mRNAs consisting of 26 identical codons with Tikhonov regularization. a): For different values of *α*, the norm of the regularized solution $\left|\right|{\vec{x}}_{\text{out},\alpha }{\left|\right|}_{2}$ is plotted versus the residual norm $||P{\vec{x}}_{\text{out},\alpha }-{\vec{s}}_{\text{sm}}{\left|\right|}_{2}$. The log-log plot shows the characteristic L-shaped curve. The optimal regularization parameter in terms of trade-off between regularization and perturbation error is *α* = 0.01 and is found by locating the corner of the curve. b): Input and fitted IFI vectors for three different regularization parameter values.

Fig 5a) shows an example of the L-curve for the decomposition of the fluorescence signature of an mRNA that consists of 26 identical codons. For three different choices of *α*, the corresponding fitted IFI vectors ${\vec{x}}_{\text{out},\alpha }$ are shown in Fig 5b), together with the random input IFI vector ${\vec{x}}_{\text{in}}$. For all choices, Tikhonov regularization led to much more reliable fit results compared to the standard least-square approach (Fig 3b)). However, high-frequency features of the input vector are filtered out and thus cannot be recovered by the fitting. For small regularization parameters (see *α* = 0.005 in Fig 5b)), the perturbation error increases and oscillatory sign changes appear. Nonetheless, the fitted IFI values are still smoother than the input values. Because of this effect, the fit quality depends on the smoothness of the input IFI values. Furthermore, for the same reasons as discussed above for the decomposition of fluorescence signatures of 4-codon-mRNAs, IFI values corresponding to the first and the last states of the translation process are fitted best, see also Fig D in S1 Text.

Additional simulation results can be found in Figs F—S in S1 Text and Figs A—H in S2 Text for a) the decomposition of fluorescence signatures of mRNAs with different lengths, b) translation processes with uniform and non-uniform translation rates, and c) processes where an unknown kinetic rate is determined simultaneously with the unknown IFI vector.

### Decomposition of experimentally determined fluorescence signatures of LepB mRNA translation

In this paragraph, we apply our method to fluorescence signatures of LepB mRNA translation. As the kinetics of LepB mRNA translation are not known at codon resolution, the simultaneous fitting of unknown kinetic rates and IFI values is necessary (see *Determination of kinetic rate ω*_45_ in S1 Text for more details). To this end, we follow an approach introduced previously for the analysis of the translation of short poly(U) mRNAs [8]: Translation of truncated mRNAs (denoted here as LepB2 to LepB11) of increasing length in terms of codons is monitored *via* fluorescent molecules that are attached to the N-termini of the nascent peptide chains and move along the *Escherichia coli* ribosomal exit tunnel. The resulting fluorescence signatures are decomposed, where for short mRNAs the simultaneous fitting of kinetic rates and IFI values yields a unique solution. In contrast, for longer mRNAs the analysis gets ambiguous (see S2 Text) and requires to take into account additional information. A longer mRNA—e.g. LepB11—cannot be directly decomposed to reveal the IFI values together with the kinetics of the process. A step-by-step analysis of the translation of increasingly longer mRNAs is required, where the results obtained for a specific mRNA can be retained for each subsequent analysis.

**Fig 6:**
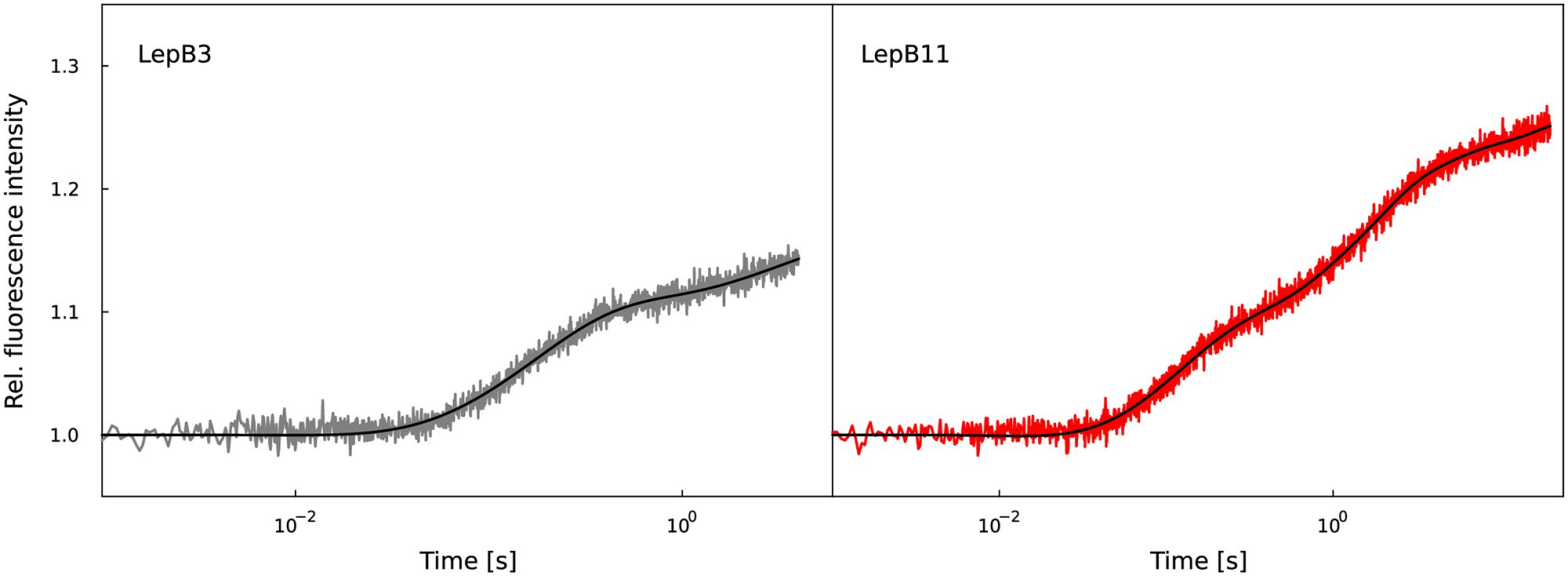
Decomposition of fluorescence signatures of LepB mRNAs. Measured fluorescence signatures of the *in-vitro* translation of LepB3 and LepB11 mRNA (colored lines) and best fits in terms of least squares (black lines).

**Fig 7:**
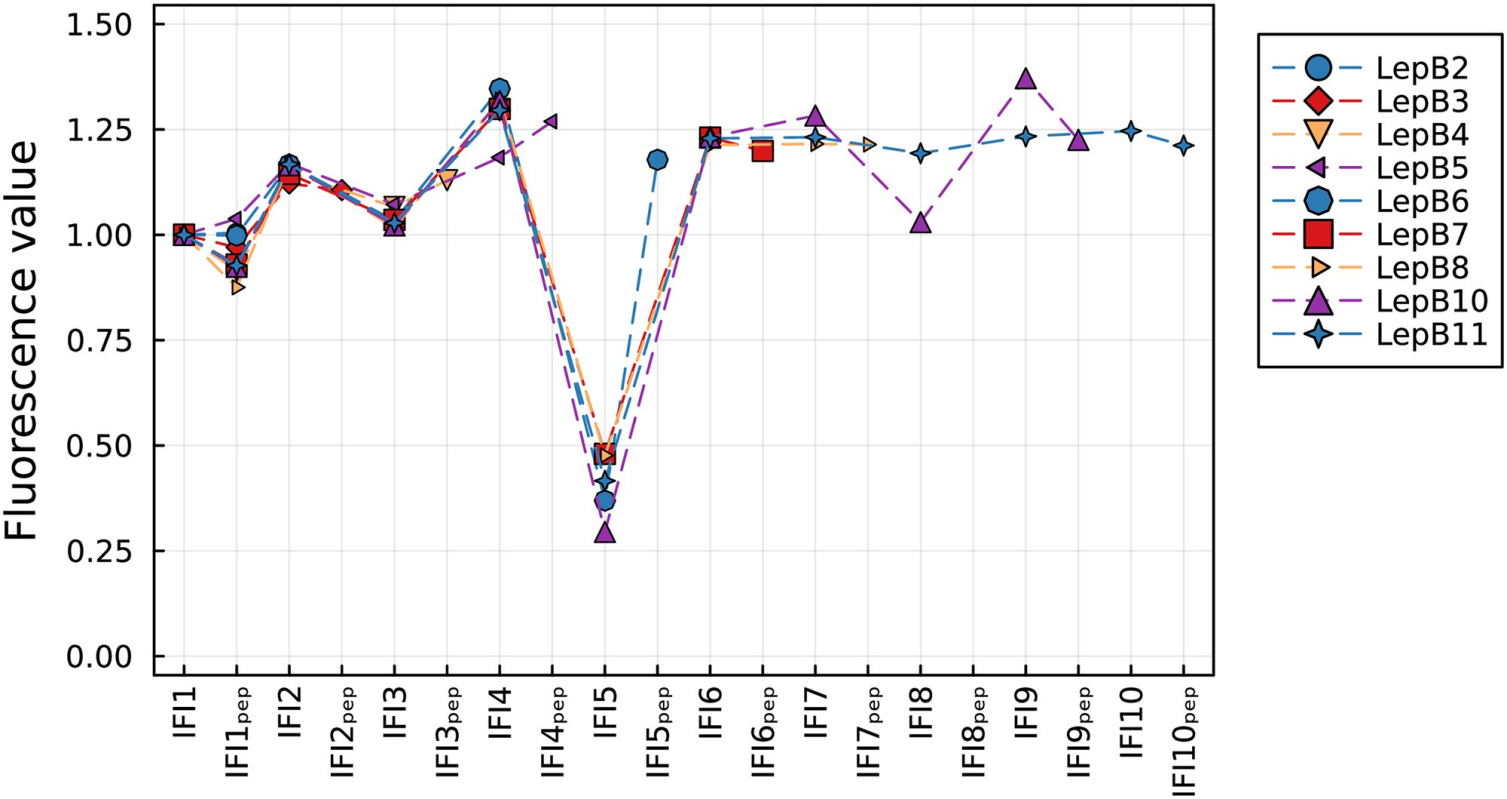
Fitted IFIs obtained from the analysis of the fluorescence signatures of truncated LepB mRNAs of different lengths. The IFIs correspond to the different states of the translation process (see Fig 8 and [8]). The lowest IFI (IFI5) is associated to the states after binding of Phe and before binding of the second Ala to the nascent peptide chain. The IFIs associated to the artificial state of a ribosome at the end of a truncated mRNA are not shown.

**Fig 8:**
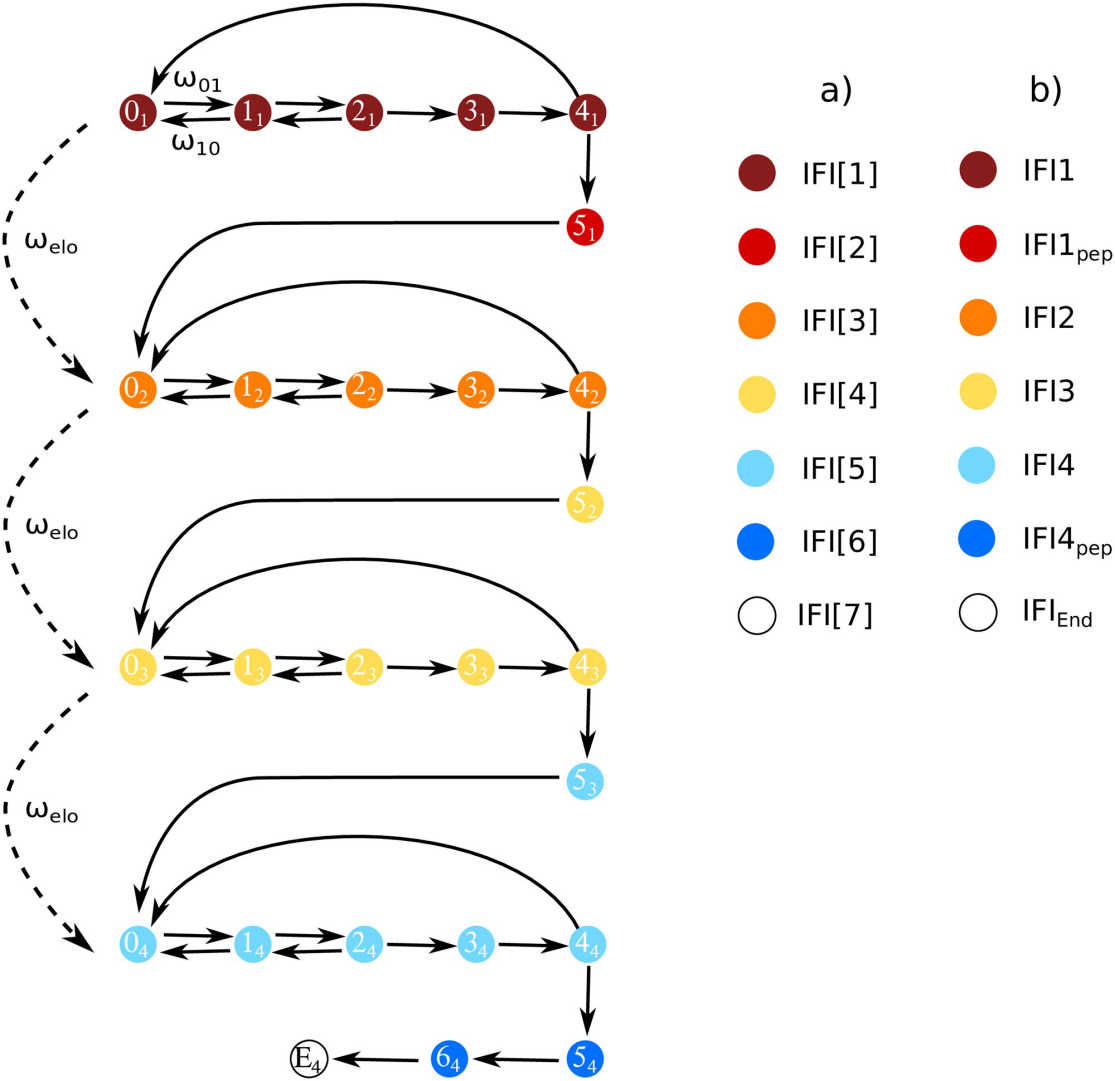
Representation of mRNA translation elongation as a Markov process and assigned IFIs for a mRNA that consists of four codons. The Markov model for translation of a short mRNA consisting of *n* = 4 codons includes 6*n* + 2 = 26 states and *n* + 3 = 7 assigned IFIs. Each state in the process corresponds to one biochemically resolved step: State 0_1_ represents the initiation complex with the start codon in the ribosomal A site. States 0_1_-4_1_ describe initial selection, including ternary complex binding and recognition, GTPase activation, GTP hydrolysis and rearrangement of EF-Tu. This is followed by tRNA accommodation and peptide bond formation (5_1_). Afterwards, the ribosome translocates to the second codon (0_2_). The elongation cycle is repeated for the next three codons before the ribosome reaches the end of the truncated mRNA (E_4_) where the P site is occupied by the fourth codon while the A site remains empty. See refs. [39–45] for more details on the translation process. Possible transitions between the states are indicated by arrows and occur with transitions rates *ω*_*ij*_, which is exemplified by the ternary complex binding rate *ω*_01_ and the unbinding rate *ω*_10_. The average rate of translation for one codon is denoted by *ω*_elo_. For the first codon, changes in the state-specific IFIs are assumed to occur both after peptide bond formation and translocation, and states assigned with identical IFI values have the same color. Note that due to initial synchronization of ribosomes, the two IFI changes on the first codon become visible in the ensemble fluorescence signature. For all following codons only one change in fluorescence intensity after peptide bond formation (after transition from state (4_*k*_) to state (5_*k*_)) is considered to avoid overfitting. The final intrinsic fluorescence intensity E_4_ corresponds to the artificial state of the ribosome at the end of the truncated mRNA. Previously published as the 6*n*2/*n*3 model in [8]. Nomenclature of IFIs for a): a generic mRNA, and b): the specific case of LepB5 mRNA.

As an example, Fig 6 shows the measured and fitted fluorescence signatures of translation for LepB3 and LepB11 (see Figs I to K in S2 Text for all nine analyzed mRNAs). During fluorescence signature decomposition, Tikhonov regularization was applied for mRNAs with five or more codons. The fitted intrinsic fluorescence intensities of the different states are depicted in Fig 7. The IFI that belongs to the translation states between binding of Phe and binding of the second Ala is considerably lower than all other IFI values (IFI5, see Fig 8b) for definition). The corresponding fitted kinetic rates are listed in Table 1. Notably, within the initial five codons (after the start codon), the translation process exhibits a significant slowdown at the fourth codon (Phe). This observation aligns with our earlier findings derived from the analysis of short poly(U) mRNA translation [8]. Furthermore, codons coding for Ile are the slowest in the studied mRNAs (positions 7 and 10), and codons coding for identical amino acids have very similar elongation rates (positions 1 and 5, 6 and 8). Together with our previous study [8], these results indicate that both the position of the codon and the encoded amino acid influence the translation rate.

**Table 1:** Codon position-specific translation rates calculated from fitted *in-vitro* rates.

Peptide	Last amino acid	Codon position (after start codon)	Codon position-specific elongation rate *ω*_elo_ [s^−^]
LepB2	A	1	4.5 ± 0.3
LepB3	N	2	3.3 ± 0.7
LepB4	M	3	2.1 ± 0.4
LepB5	F	4	0.8 ± 0.3
LepB6	A	5	4.5 ± 0.4
LepB7	L	6	0.9 (- 0.5 + 0.8)
LepB8	I	7	0.6 (- 0.5 + 1.6)
n/a	L	8	1*
LepB10	V	9	1.2 ± 0.6
LepB11	I	10	0.2 (- 0.1 + 0.9)

For short mRNAs, simultaneously fitting kinetic rates and IFI values results in a unique solution, while the analysis gets more ambiguous for longer mRNAs (see Figs L and M in S2 Text). “Peptide” indicates which peptide is encoded by the mRNA that was analyzed to determine the reported codon position-specific translation rate. The second column states the last amino acid of the peptide, which is encoded by the codon at the indicated position in LepB mRNA.

*: The translation rate *ω*_elo,8_ of the eighth codon (after the start codon) is not directly fitted from a data curve (n/a) but inferred based on reasonable IFI values for subsequent analysis. For detailed information see Table B in S2 Text.

## Discussion

We presented a least-squares approach to decompose time-dependent ensemble signals of initially synchronized processive enzymatic reactions and analyzed the limits of the method. The decomposition allows to identify state-specific signal intensities for the individual steps of a reaction, which can be valuable information already by itself or part of a larger model-fitting procedure [8]. To demonstrate the method with a practical example, we modeled mRNA translation as a Markov process and assigned a specific intrinsic fluorescence intensity (IFI) to each state in the Markov chain. We computed the time-dependent fractions of ribosomes in the different states of the translation process and determined the corresponding ensemble fluorescence signatures of mRNA translation, including thermal noise. These simulated ensemble fluorescence signatures were then used to test our decomposition method by comparison of input and fitted output IFI values. Additionally, we applied our method to decompose experimental fluorescence signatures of LepB mRNA translation. We found that for reactions with low processivity (corresponding to seven different IFI values in our mRNA translation example), a standard least-squares approach is sufficient to reliably fit IFI values with good precision. In contrast, investigation of model properties in terms of singular values and condition numbers revealed clear signs of ill-posed problems for reactions with higher processivity. However, by using least-square fitting with Tikhonov regularization it is still possible to obtain meaningful solutions. From these observations we draw the conclusion that even before a bulk experiment with initially synchronized processive enzymes is conducted, the following steps can be taken to assess the feasibility of ensemble signal decomposition: First, a model that appropriately describes the stochastic biochemical process of interest needs to be developed, including the assignment of IFIs to the different states of the model. Second, based on this model, the matrix of state occupancy probabilities and its condition number have to be computed (see Eq (4) and S2 Text). For small condition numbers, a standard least-squares approach might be sufficient to decompose ensemble time traces, given that no other factors, such as a low signal-to-noise ratio, prevent a meaningful analysis of the data. Otherwise, regularization is required or, alternatively, strategies must be developed to help decrease the condition number, including, for example, additional measurements to reduce the number of free parameters. Bulk experiments are often much more cost- and time-efficient than methods probing single molecules directly, but they lack the depth of information that the latter approaches achieve. Decomposition of ensemble signals can expand the range of bulk methods by providing a way to extract more detailed information from the same experimental setup. For example, analysis of the codon-by-codon translation of LepB mRNA presented here demonstrates the differences of elongation rates at different codons and supports the notion that translation rate drops at the fourth translated codon (i.e., the last codon of LepB5 mRNA), consistent with earlier data [8]. We also observe slow translation at positions 7 and 10. The reasons for the observed slow down are unknown and may be related to both translation *per se* or protein folding in the narrow exit tunnel of the ribosome, which may regulate the translation rate. Of note, the stalling codons at positions 7 and 10 encode for Ile, which is one of the amino acids prevalent in inhibitory bicodons [31]. These results in combination with our previous findings [8] show that both the codon position and the encoded amino acid have an effect on the elongation rate. Also IFI analysis reveals an anisotropic environment of the N-terminal reporter group, which in the future can be used to study the effect of individual mutations in the peptide or mutations in the ribosome, e.g., on translational rates or co-translational protein folding. Our approach extends beyond the decomposition of fluorescence signatures in mRNA translation and can be applied to assess various bulk signals originating from biological processes. To achieve this, a prerequisite is a comprehensive understanding of the underlying process, including its potential states and the kinetic rates governing transitions between these states. The subsequent conscientious assignment of state-specific fluorescence intensities ensures biological relevance throughout the analysis.

## Materials and methods

### Modeling of mRNA translation as a Markov process

In the literature, different approaches are used for the modeling of mRNA translation and the determination of kinetic rates ([15, 32–36]). In this work, we modeled mRNA translation as a time-continuous Markov process as is described in ref. [8, 37], and [38]. For the sake of consistency both in terms of modeling approach and model parameterization, like in our earlier work, the Markov model used here describes a large fraction of the translation-elongation cycle steps that were biochemically resolved and of which the corresponding kinetic rates were investigated by Rodnina and co-workers over the past decades [39–45]. Fig 8 shows as an example the Markov model for translation of an mRNA that consists of four codons. By solving the master equations of the Markov process

$$\begin{array}{c} \frac{d}{dt}P_{i}\left(t\right)=\sum_{j=1}^{N}P_{j}\left(t\right)\;\omega_{ji}-P_{i}\left(t\right)\;\omega_{ij} \end{array}$$ (3)

the time evolution of the occupancy probability *P*_i_(*t*), which is the probability to find a ribosome in state *i* = 1, …*N* at time *t*, is obtained for a given set of transition rates *ω*_*ij*_ from state *i* to state *j* (see Table 2). The average rate of translation *ω*_elo_ for one codon can be calculated by first step analysis [46] using the transition rates *ω*_*ij*_ [37]. According to the experimental setup in [8], the translation processes are initially synchronized at state *i* = 0 at time *t* = 0, i.e., the initial condition is

$$\begin{array}{c} P_{0}\left(0\right)=1\;\text{and}\;P_{i}\left(0\right)=0\;\text{for}\;\text{all}\;i\neq 0. \end{array}$$

**Table 2:** Transition rates.

Rate	Value at 37°C
*ω*_01_	350 s^−1^
*ω*_10_	700 s^−1^
*ω*_12_	1500 s^−1^
*ω*_21_	2 s^−1^
*ω*_23_	1500 s^−1^
*ω*_34_	450 s^−1^
*ω*_40_	1 s^−1^
*ω*_45_	13.8 s^−1^
*ω*_50_	53 s^−1^
*ω*_end_	0.3 s^−1^
*ω*_elo_	10 s^−1^

Apart from the rates *ω*_45_, *ω*_50_ and *ω*_end_ all transition rates *ω*_*ij*_ are as used in the Markov model description of the translation process in [37]. The average rate of translation *ω*_elo_ for one codon is calculated by first step analysis [37]. The rate *ω*_end_ describes the transition into the artificial end state of the ribosome at the end of the truncated mRNA.

The matrix of occupancy probabilities **P** is defined as

$$\begin{array}{c} P=(\begin{array}{cccc} P_{0}\left(t_{1}\right) & P_{1}\left(t_{1}\right) & \cdots & P_{N}\left(t_{1}\right)\\ P_{0}\left(t_{2}\right) & P_{1}\left(t_{2}\right) & \cdots & P_{N}\left(t_{2}\right)\\ \vdots & \vdots & \ddots & \vdots \\ P_{0}\left(t_{M}\right) & P_{1}\left(t_{M}\right) & \cdots & P_{N}\left(t_{M}\right) \end{array}), \end{array}$$ (4)

where *N* is the number of states in the Markov process and *M* is the number of considered time points. The time points of the simulation were selected based on the experimental temporal resolution [8].

### Generation and fitting of test data

**Fig 9:**
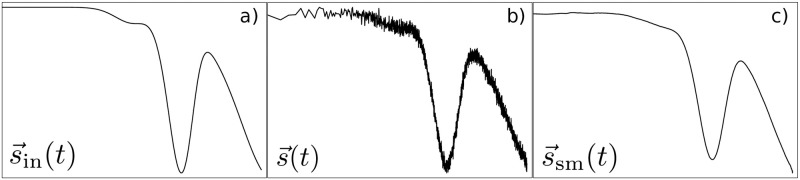
Generation of simulated data. a): Simulated ensemble fluorescence signature ${\vec{s}}_{\text{in}}\left(t\right)$ constructed from the random IFI input vector ${\vec{x}}_{\text{in}}$ weighted by the time-dependent state occupancy probabilities **P** of the Markov process. b): Superimposition of Gaussian noise to imitate thermal fluctuations. c): The simulated data curve $\vec{s}$ is smoothed using a moving average. Note: To make differences in ${\vec{s}}_{\text{in}}\left(t\right)$ and ${\vec{s}}_{\text{sm}}\left(t\right)$ visible, a Gaussian noise with a standard deviation of 0.01 and a moving average with a non-optimal subset size of 200 steps were used in this figure.

The random IFI input vector ${\vec{x}}_{\text{in}}$ is created using the MATLAB function rand() for uniformly distributed random numbers as follows: ${\vec{x}}_{\text{in}}=2\cdot \text{rand}\left(n,1\right)$, where *n* is the number of required IFI components. The values of the IFI input vector range between 0 and 2, where the first IFI is set to 1. The simulated ensemble fluorescence signature ${\vec{s}}_{\text{in}}\left(t\right)$ contains *m* = 1...*M* fluorescence values, one for each time point *t*_*m*_. Each individual component of ${\vec{s}}_{\text{in}}\left(t\right)$ is given by the sum over all components of the IFI input vector ${\vec{x}}_{\text{in}}$ weighted by the time-dependent state occupancy probabilities **P** of the Markov process (see Eqs (1) and (4)). The simulated fluorescence signature ${\vec{s}}_{\text{in}}\left(t\right)$ is superimposed with a Gaussian random noise $\vec{\Sigma }$ with a standard deviation of 0.005. The influence of the chosen standard deviation on the fitted IFI vector ${\vec{x}}_{\text{out}}$ is shown in Fig E in S1 Text. The time points in the simulation and the random noise levels are based on experimental data [8]. The data are smoothed using the MATLAB function smooth() with the specified method moving and a subset size of 30 data points to obtain ${\vec{s}}_{\text{sm}}\left(t\right)≔smooth\left(\vec{s}\left(t\right)\right)$. See Fig 9 for an exemplary representation of ${\vec{s}}_{\text{in}}\left(t\right)$, $\vec{s}\left(t\right)$, and ${\vec{s}}_{\text{sm}}\left(t\right)$. We use the non-negative least-squares solver lsqnonneg in MATLAB to solve the system of linear Eq (2) with the components of the IFI vector ${\vec{x}}_{\text{out}}$ as adjustable parameters.

### Buffer and reagents

All experiments were carried out in HiFi buffer (50 mM Tris-HCl, pH 7.5, 70 mM NH 4Cl, 30 mM KCl and 3.5 mM MgCl_2_, 8 mM putrescine, 0.5 mM spermidine, and 1 mM DTT) [47]. Chemicals were from Roche Molecular Biochemicals, Sigma Aldrich or Merck, nucleotide triphosphates from Jena Bioscience. Radioactive compounds were from Hartmann Analytic. Total *E. coli* tRNA was from Roche. EF-Tu, initiation factors, [3H]Met-tRNA fMet, and Bodipy-[3H]Met-tRNA fMet were prepared from *E. coli* as described [47]. 70S ribosomes were prepared from *E. coli* according to the published protocol [48]. 70S initiation complexes were prepared by incubating 70S ribosomes (typically 0.5 μM) with a 1.5-fold excess of IF1, 2, and 3, 1.5-fold excess of Bodipy-[3H]Met-tRNA fMet, and a 4-fold excess of the mRNA of the indicated length in the presence of 1 mM GTP, 3 mM phosphoenolpyruvate and 1 μg μL^-1^ pyruvate kinase for 15 min at 37°C. Translation was performed in the stopped-flow apparatus by mixing 70S initiation complexes (0.025 μM final concentration) with EF-Tu (15 μM), purified total aminoacyl-tRNA (40 μM) and EF-G (0.05 μM) in the presence of 1 mM GTP, 3 mM phosphoenolpyruvate and 1 μg μL^-1^ pyruvate kinase. The fluorophore Bodipy was exited at 470nm and the emission monitored after passing a 500 nm cut-off filter. The time courses are averages of 6–8 technical replicates.

## Supporting information

S1 Text File containing additional simulation results. (PDF)

S2 Text File containing additional simulation results and further supplementary information. (PDF)
