# Supplementary material for: Decomposing bulk signals to reveal hidden information in processive enzyme reactions: A case study in mRNA translation
Source: PLoS Comput Biol. 2024 Mar 5;20(3):e1011918. doi: 10.1371/journal.pcbi.1011918 (PMC10942256; doi:10.1371/journal.pcbi.1011918)
Supplement: S1 Text — (PDF) [file pcbi.1011918.s001.pdf]

Supplemental Information

Decomposing bulk signals to reveal hidden information in processive enzyme reactions: A case study in mRNA translation

Nadin Haase[1], Wolf Holtkamp[2,3], Simon Christ[1], Dag Heinemann[4,5,6], Marina V. Rodnina[2], and Sophia Rudorf[1,*]

[1]Leibniz University Hannover, Institute of Cell Biology and Biophysics, Germany
[2]Max Planck Institute for Multidisciplinary Sciences, Department of Physical Biochemistry, Germany
[3]Paul-Ehrlich-Institut, Division of Allergology, Germany.
[4]Leibniz University Hannover, Hannover Centre for Optical Technologies (HOT), Germany
[5]Leibniz University Hannover, Institute of Horticultural Production Systems, Germany
[6]Leibniz University Hannover, PhoenixD Cluster of Excellence, Germany
[*]rudorf@cell.uni-hannover.de

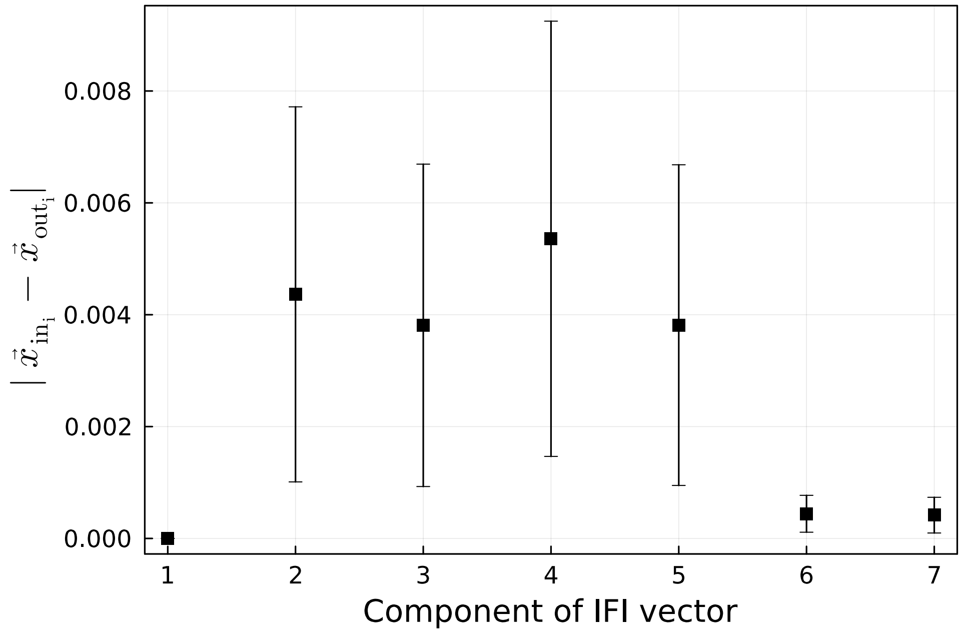

Figure A: **Component-wise deviation between fitted ($\vec{x}_{\mathrm{out}}$) and given IFI vector ($\vec{x}_{\mathrm{in}}$) obtained from the analysis of the fluorescence signatures of an mRNA that consists of four identical codons.** The shown data is an average for the decomposition of fluorescence signatures simulated from 1000 random input IFI vectors. The first entry of the IFI vector is always set to 1. IFI values related to the last states in Fig. 6 in the main text are fitted with higher reliability.

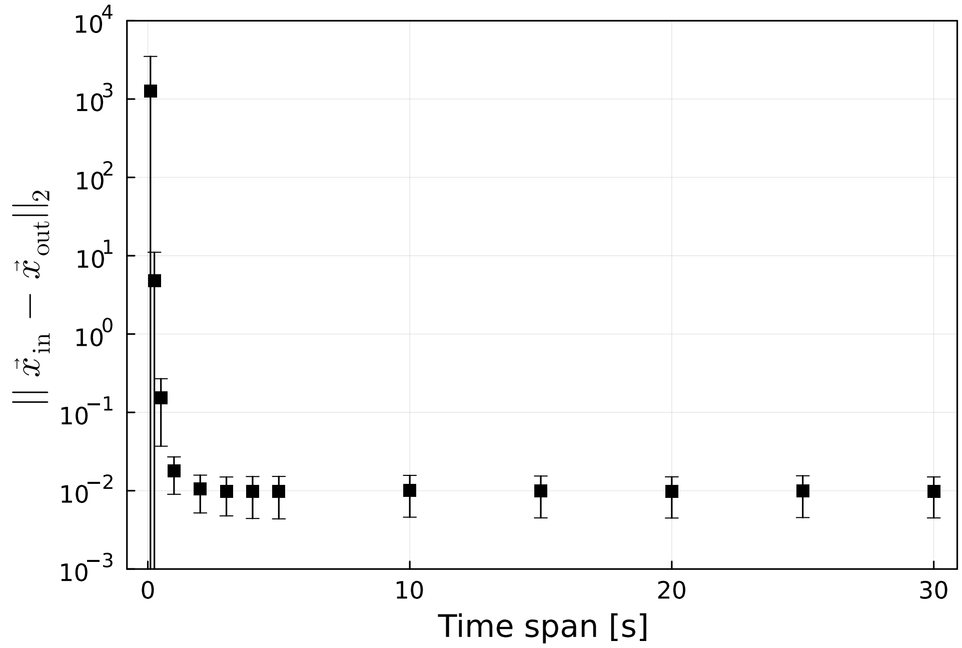

Figure B: **Deviation between fitted ($\vec{x}_{\mathrm{out}}$) and given IFI vector ($\vec{x}_{\mathrm{in}}$) obtained from the analysis of the fluorescence signatures of an mRNA that consists of four identical codons.** The shown data are averages of the decomposition of fluorescence signatures simulated from 1000 random input IFI vectors. The process was evaluated for different time spans.

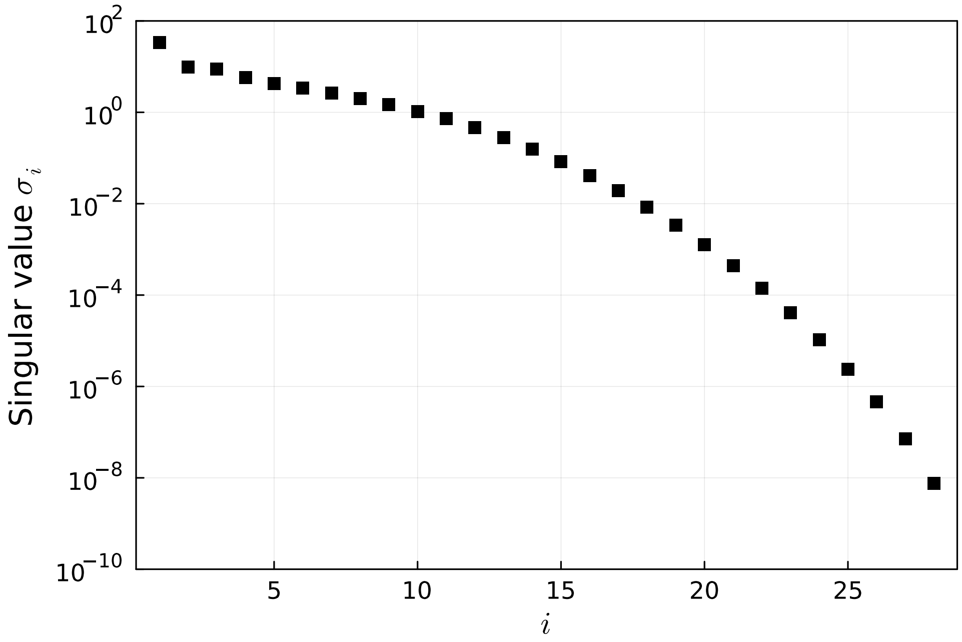

Figure C: **Singular values σ_i of the occupancy probabilities matrix P for translation of an mRNA consisting of 26 codons.** The singular values decay gradually to zero without jumps between consecutive singular values.

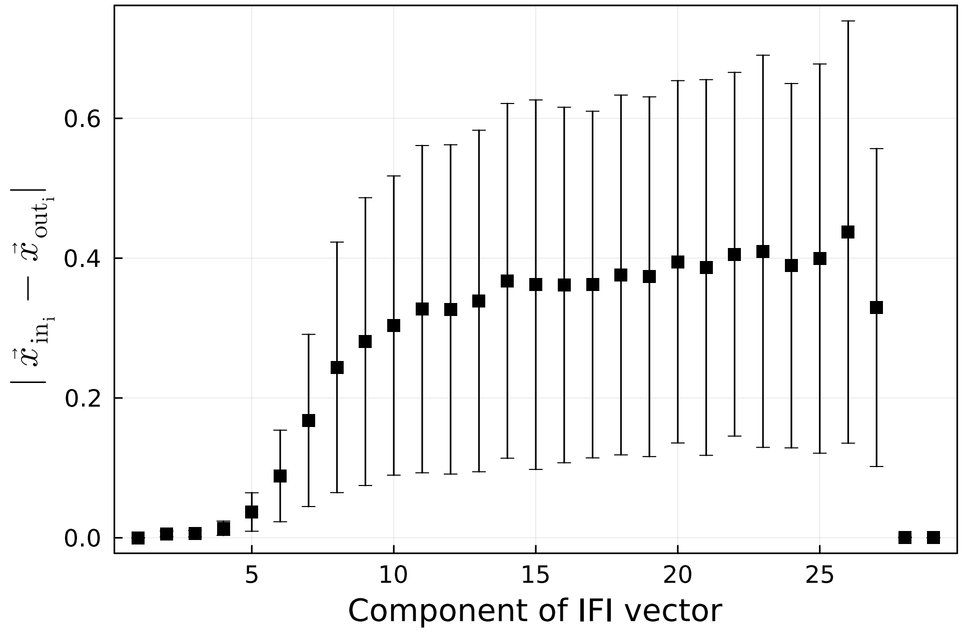

Figure D: **Component-wise deviation between fitted ($\vec{x}_{\mathrm{out}}$) and given IFI vectors ($\vec{x}_{\mathrm{in}}$) obtained from the analysis of fluorescence signatures of mRNAs that consist of 26 identical codons.** The shown data are averages of the decomposition (with Tikhonov regularization) of fluorescence signatures simulated from 1000 random input IFI vectors. IFI values corresponding to the first and last states of the translation process are fitted with higher accuracy.

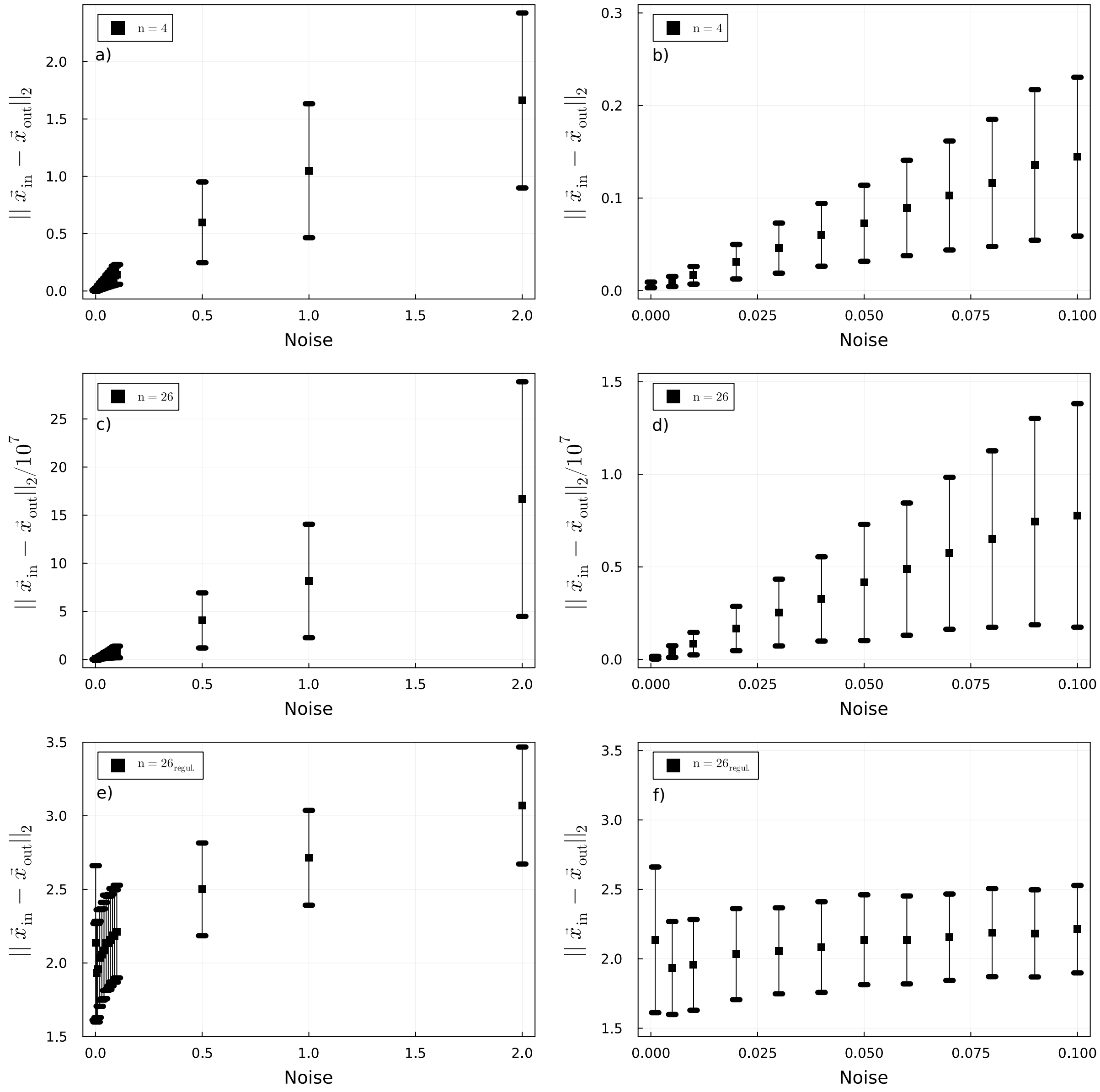

Figure E: **The deviation between fitted ($\vec{x}_{\text{out}}$) and given IFI vector ($\vec{x}_{\text{in}}$) is plotted versus the standard deviation of the superimposed Gaussian noise $\vec{\Sigma}$.** The data are obtained from the analysis of fluorescence signatures of mRNAs that consist of four identical codons (a and b), of 26 identical codons (c and d) and of 26 identical codons using Tikhonov regularization (e and f). Shown are averages for 1000 random input IFI vectors. Figures on the right side zoom into the range of small standard deviations.

Determination of kinetic rate ω_{45}

We tested the robustness of our method by systematically using rates for the fit that are not the same as the rates used to generate the data and observing the effects on the fitted results. To perform this analysis, a for-loop is introduced into the fitting procedure that systematically changes the kinetic rate (in our case ω_{45}). For each iteration, the best theoretical fit in terms of least squares is saved. Once the for-loop is completed, the kinetic rate is found by identifying the minimum goodness-of-fit parameter χ^2 [1], which represents a measurement for the deviation of the simulated and calculated data curve:

$$\chi^2 = \sum \left\{ \frac{1}{\sigma^2} [\mathbf{P}\vec{x}_{\text{out},\alpha} - \vec{s}_{\text{sm}}]^2 \right\}$$

The standard deviation σ is used as an estimation for the error in the smoothed data curve. An increase in χ^2 by 1 results in an increase of 1 standard deviation σ for the parameter [1]. We performed the simulation for uniform and non-uniform translation elongation rates.

4-codon-mRNA - uniform input rates

All transition rates apart from ω_{45} of the Markov model description of the translation process are used as stated in Table 2 in the main paper. The rate ω_{45} is set to $\omega_{45_{\text{input}}} = 14\ \text{s}^{-1}$ for all codons and the fitting procedure to identify the unknown IFIs is performed as usual with the kinetic rate ω_{45} as additional parameter. Figure F shows the results of the decomposition. The best theoretical fit is found for $\omega_{45_{\text{fit}}} = 14\text{s}^{-1}$ (see Fig. G), which is in agreement with the input parameter.

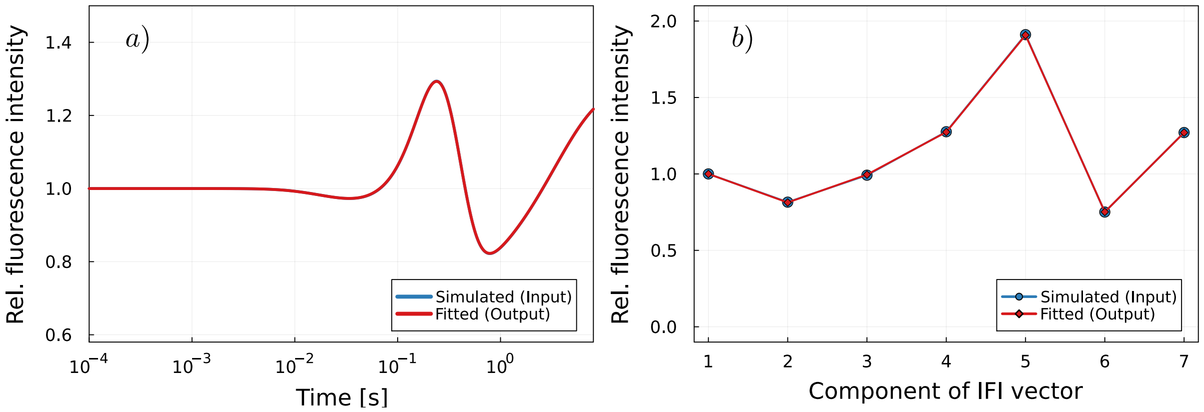

Figure F: **Fluorescence signature of an mRNA that consists of four identical codons for random IFI input vector.** a): The simulated fluorescence signature is compared to the best theoretical fit in terms of least squares. The theoretical model and simulated data curves are in perfect agreement. b): Fitted IFIs obtained from the analysis of the fluorescence signature compared to the given IFI input vector.

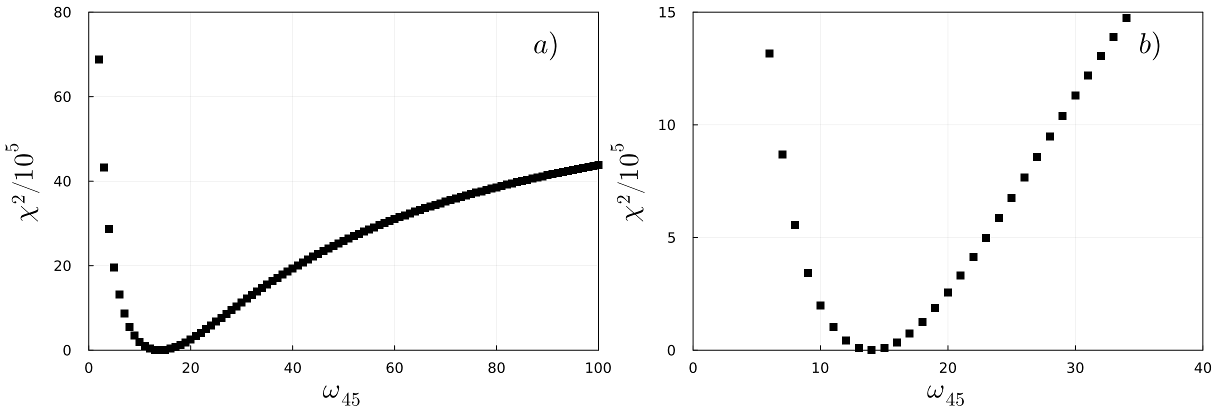

Figure G: **Determination of kinetic rate ω_{45} from fluorescence signature of an mRNA that consists of four identical codons for random IFI input vector.** a): The goodness-of-fit parameter χ^2 is calculated for different values of the unknown kinetic rate ω_{45}. The kinetic rate with the minimal χ^2 is identified. b): Enhanced depiction of the local minimum which is found for $\omega_{45_{\text{fit}}} = 14\text{s}^{-1}$, which is in agreement with the input parameter $\omega_{45_{\text{input}}} = 14\ \text{s}^{-1}$.

4-codon-mRNA - non-uniform input rates

All transition rates apart from ω_{45} of the Markov model description of the translation process are used as stated in Table 2 in the main paper. The rate ω_{45} is set to different values for each codon, see Table A. The fitting procedure to identify the unknown IFIs is performed as usual with the kinetic rate ω_{45_4} as additional parameter. Figure H shows the results of the decomposition. The best theoretical fit is found for $\omega_{45_4,\text{fit}} = 14\text{s}^{-1}$ (see Fig. I), which is in agreement with the input parameter.

Table A: For each codon, a different rate for ω_{45_x} is used in the Markov model description of the translation process resulting in a non-uniform translation elongation rate. $^*\omega_{45_4}$ is assumed to be the unknown kinetic rate to be fitted.

Rate	Input value
ω_{45_1}	10 s^{-1}
ω_{45_2}	2 s^{-1}
ω_{45_3}	45 s^{-1}
$\omega^*_{45_4}$	14 s^{-1}

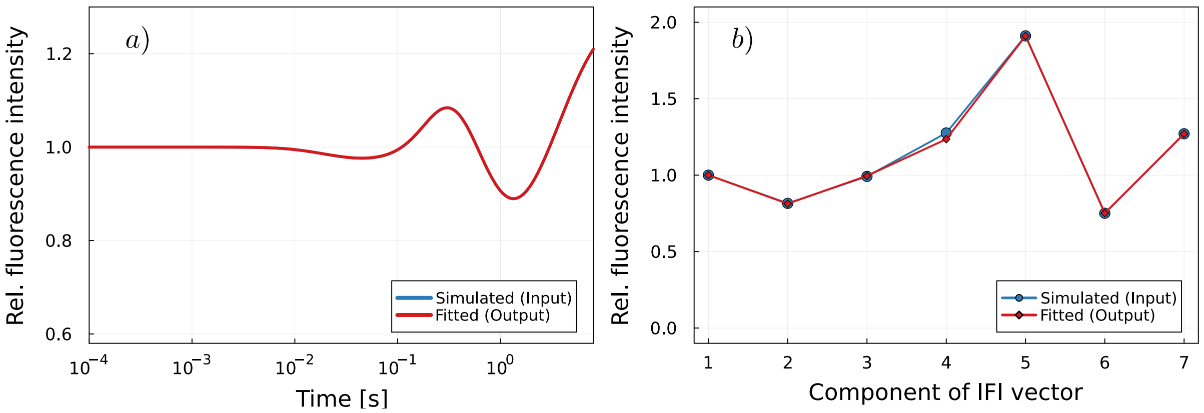

Figure H: **Fluorescence signature of an mRNA that consists of four codons with non-uniform translation rates for random IFI input vector.** a): The simulated fluorescence signature is compared to the best theoretical fit in terms of least squares. The theoretical model and simulated data curves are in perfect agreement. b): Fitted IFIs obtained from the analysis of the fluorescence signature compared to the given IFI input vector.

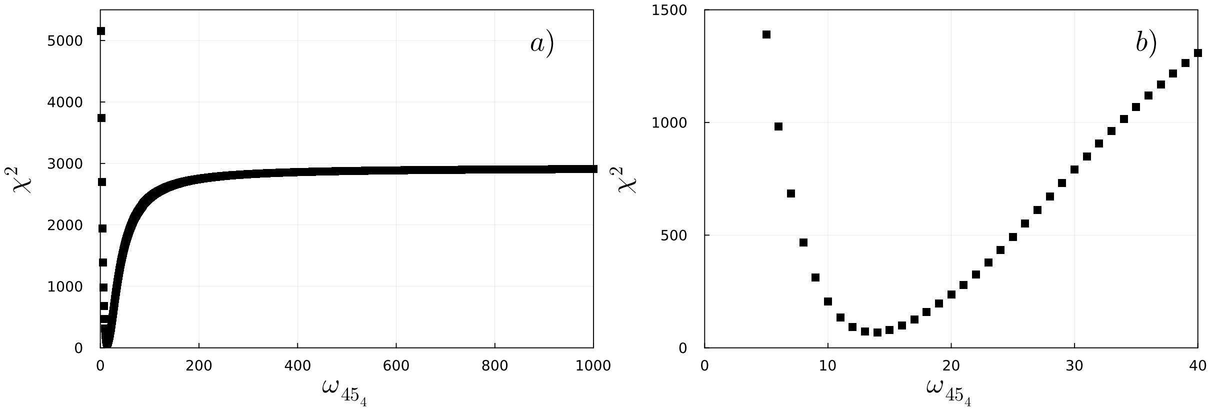

Figure I: **Determination of kinetic rate ω_{45_4} from fluorescence signature of an mRNA that consists of four codons with non-uniform translation rates for random IFI input vector.** a): The goodness-of-fit parameter χ^2 is calculated for different values of the unknown kinetic rate ω_{45_4}. The kinetic rate with the minimal χ^2 is identified. b): Enhanced depiction of the local minimum which is found for $\omega_{45_4,\text{fit}} = 14\text{s}^{-1}$, which is in agreement with the input parameter $\omega_{45_4,\text{input}} = 14\text{s}^{-1}$.

26-codon-mRNA - uniform input rates

All transition rates apart from ω_{45} of the Markov model description of the translation process are used as stated in Table 2 in the main paper. The rate ω_{45} is set to $\omega_{45_{\text{input}}} = 14\ \text{s}^{-1}$ for all codons and the fitting procedure to identify the unknown IFIs is performed as usual with the kinetic rate ω_{45} as additional parameter. Figure J shows the results of the decomposition. The best theoretical fit is found for $\omega_{45_{\text{fit}}} = 14\text{s}^{-1}$ (see Fig. K), which is in agreement with the input parameter.

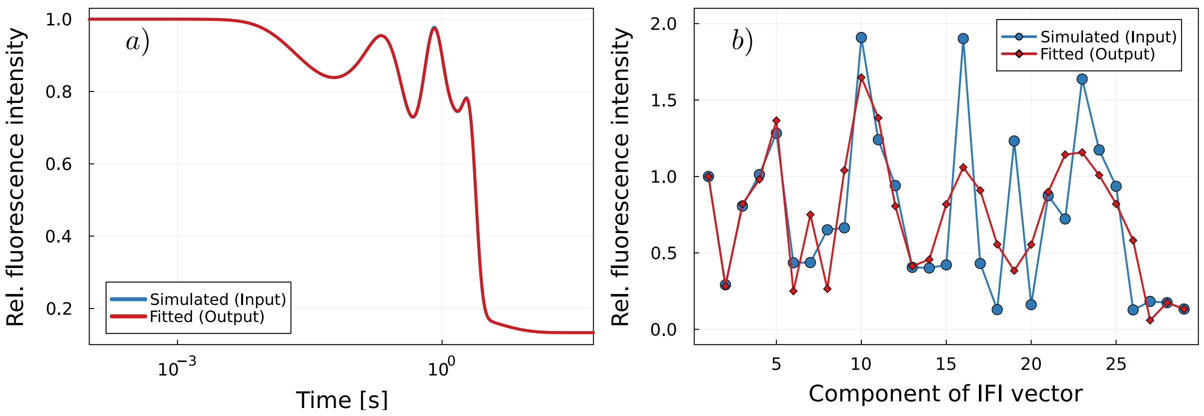

Figure J: **Fluorescence signature of an mRNA that consists of 26 identical codons for random IFI input vector.** a): The simulated fluorescence signature is compared to the best theoretical fit in terms of least squares. The theoretical model and simulated data curves are in perfect agreement. b): Fitted IFIs obtained from the analysis of the fluorescence signature compared to the given IFI input vector.

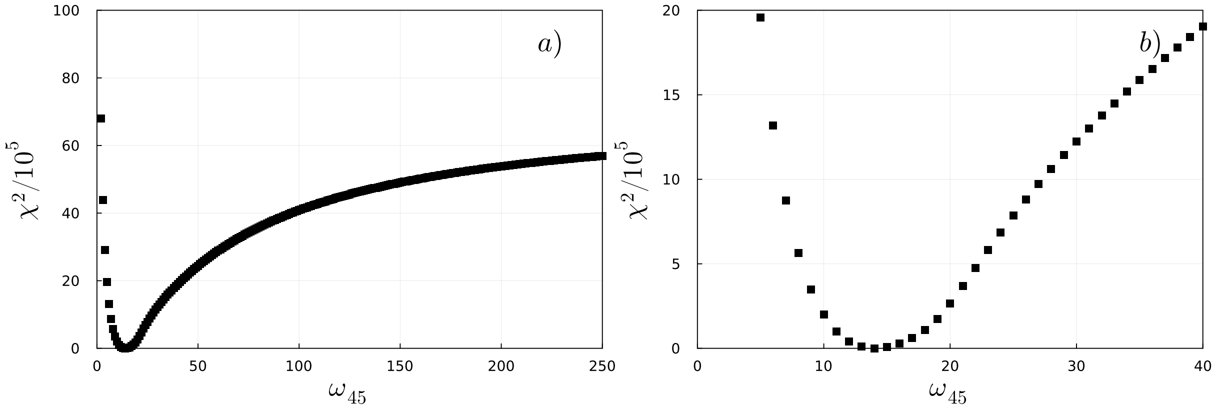

Figure K: **Determination of kinetic rate ω_{45} from fluorescence signature of an mRNA that consists of 26 identical codons for random IFI input vector.** a): The goodness-of-fit parameter χ^2 is calculated for different values of the unknown kinetic rate ω_{45}. The kinetic rate with the minimal χ^2 is identified. b): Enhanced depiction of the local minimum which is found for $\omega_{45_{\text{fit}}} = 14\text{s}^{-1}$, which is in agreement with the input parameter $\omega_{45_{\text{input}}} = 14\ \text{s}^{-1}$.

26-codon-mRNA - non-uniform input rates

Table B: For each codon, a different rate for ω_{45_x} is used in the Markov model description of the translation process resulting in non-uniform translation elongation rates. For selected codons, ω_{45_x} is determined from the fluorescence signature of an mRNA that consists of 26 codons ('-' no simulation was performed for this position). It is assumed that all kinetic rates are known except for the fitted rate. A pronounced minimum in the χ^2 curve is necessary for a unique identification of the best fit. A distinct minimum can only be found for the first four codons. The * indicates that the χ^2 curve does not deploy an optimum but rather a minimal value for the kinetic rate, see the figures below. The codon-specific elongation rate ω_{elo} is calculated by first step analysis [2] using the transition rates ω_{ij} [3].

Rate	Input ω_{45} [s^{-1}]	Fitted ω_{45} [s^{-1}]	ω_{elo} (from input ω_{45}) [s^{-1}]
ω_{45_1}	80	80	25.5
ω_{45_2}	11	14	8.5
ω_{45_3}	43	46	20
ω_{45_4}	2	2	1.9
ω_{45_5}	13	*	9.6
ω_{45_6}	15	*	10.7
ω_{45_7}	10	*	7.8
ω_{45_8}	17	*	11.6
ω_{45_9}	15	*	10.7
$\omega_{45_{10}}$	15	*	10.7
$\omega_{45_{11}}$	25	-	15
$\omega_{45_{12}}$	35	-	18
$\omega_{45_{13}}$	49	-	21.2
$\omega_{45_{14}}$	19	*	12.6
$\omega_{45_{15}}$	33	-	17.5
$\omega_{45_{16}}$	28	-	16
$\omega_{45_{17}}$	7	-	5.9
$\omega_{45_{18}}$	24	*	14.6
$\omega_{45_{19}}$	10	-	7.8
$\omega_{45_{20}}$	10	-	7.8
$\omega_{45_{21}}$	12	-	9
$\omega_{45_{22}}$	18	*	12.1
$\omega_{45_{23}}$	15	-	10.7
$\omega_{45_{24}}$	10	-	7.8
$\omega_{45_{25}}$	20	-	13
$\omega_{45_{26}}$	14	*	10.1

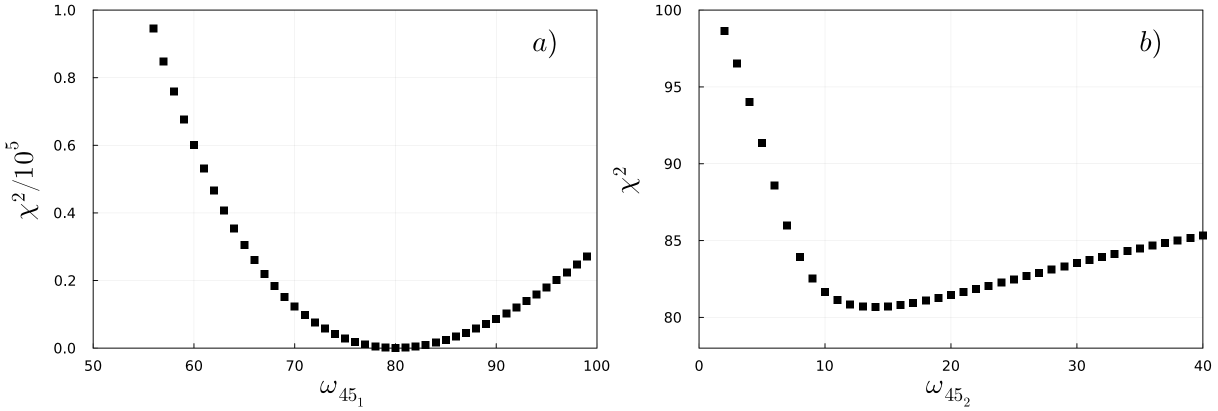

Figure L: **Determination of kinetic rates ω_{45_1} and ω_{45_2} from fluorescence signatures of mRNAs that consist of 26 codons for random IFI input vectors.** It is assumed that all kinetic rates are known except for the rate $\omega_{45_{1(2)}}$ for codon 1 (2). Plot of the goodness-of-fit parameter χ^2 versus the unknown kinetic rate a): ω_{45_1} and b): ω_{45_2}. The local minima are found at $\omega_{45_1} = 80\text{s}^{-1}$ and $\omega_{45_2} = 14\text{s}^{-1}$. The input rates used to simulate the fluorescence signatures are $\omega_{45_1} = 80\text{s}^{-1}$ and $\omega_{45_2} = 11\text{s}^{-1}$.

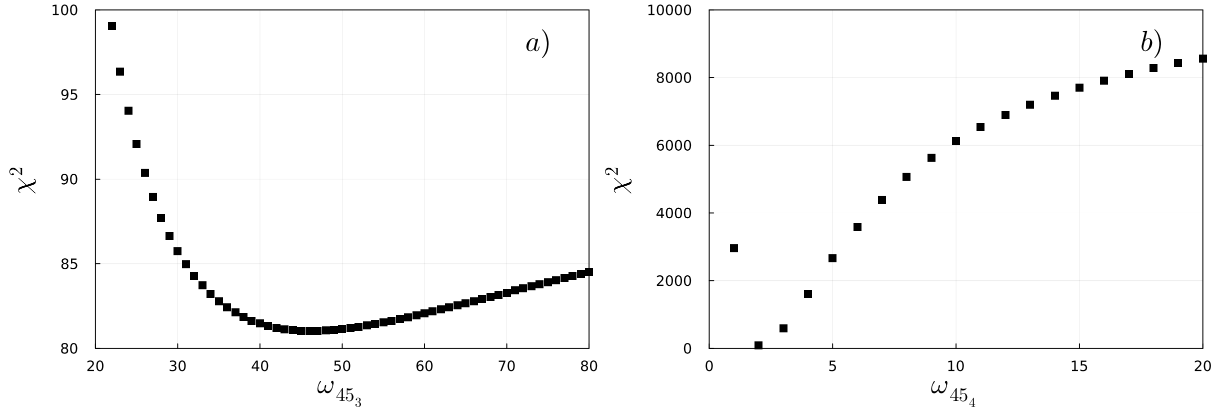

Figure M: **Determination of kinetic rates ω_{45_3} and ω_{45_4} from fluorescence signatures of mRNAs that consist of 26 codons for random IFI input vectors.** It is assumed that all kinetic rates are known except for the rate $\omega_{45_{3(4)}}$ for codon 3 (4). Plot of the goodness-of-fit parameter χ^2 versus the unknown kinetic rate a): ω_{45_3} and b): ω_{45_4}. The local minima are found at $\omega_{45_3} = 46\text{s}^{-1}$ and $\omega_{45_4} = 2\text{s}^{-1}$. The input rates used to simulate the fluorescence signatures are $\omega_{45_3} = 43\text{s}^{-1}$ and $\omega_{45_4} = 2\text{s}^{-1}$.

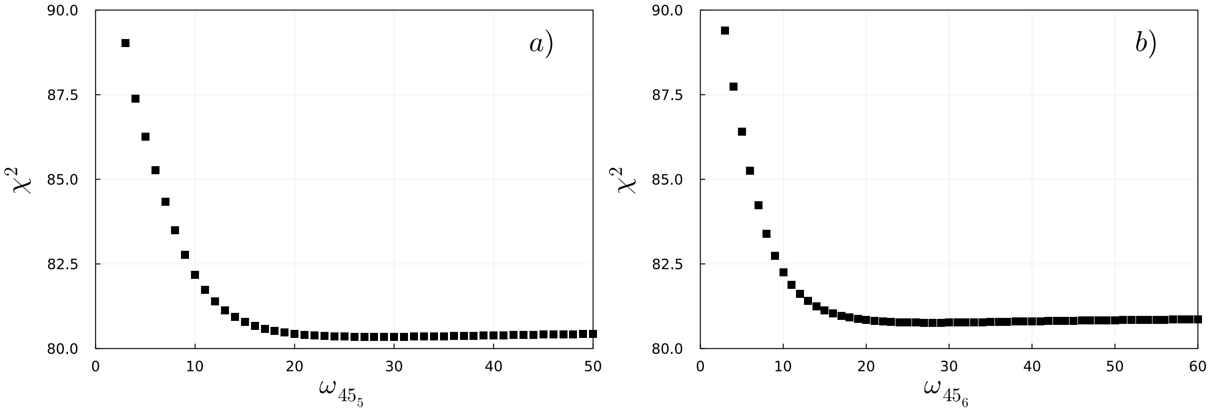

Figure N: **Determination of kinetic rates ω_{45_5} and ω_{45_6} from fluorescence signatures of mRNAs that consist of 26 codons for random IFI input vectors.** It is assumed that all kinetic rates are known except for the rate $\omega_{45_{5(6)}}$ for codon 5 (6). Plot of the goodness-of-fit parameter χ^2 versus the unknown kinetic rate a): ω_{45_5} and b): ω_{45_6}. The local minima are found at $\omega_{45_5} = 28\mathrm{s}^{-1}$ and $\omega_{45_6} = 28\mathrm{s}^{-1}$. The input rates used to simulate the fluorescence signatures are $\omega_{45_5} = 13\mathrm{s}^{-1}$ and $\omega_{45_6} = 15\mathrm{s}^{-1}$.

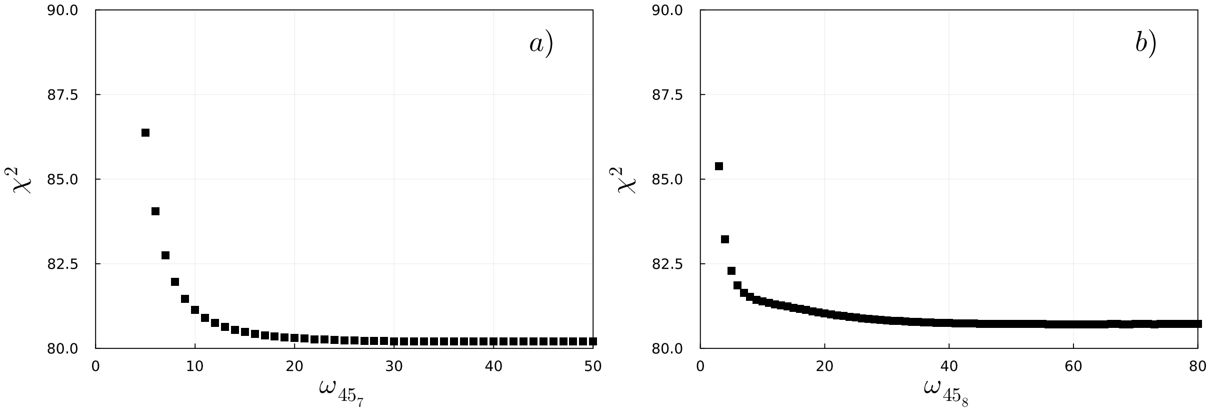

Figure O: **Determination of kinetic rates ω_{45_7} and ω_{45_8} from fluorescence signatures of mRNAs that consist of 26 codons for random IFI input vectors.** It is assumed that all kinetic rates are known except for the rate $\omega_{45_{7(8)}}$ for codon 7 (8). Plot of the goodness-of-fit parameter χ^2 versus the unknown kinetic rate a): ω_{45_7} and b): ω_{45_8}. The local minima are found at $\omega_{45_7} = 39\mathrm{s}^{-1}$ and $\omega_{45_8} = 68\mathrm{s}^{-1}$. The input rates used to simulate the fluorescence signatures are $\omega_{45_7} = 10\mathrm{s}^{-1}$ and $\omega_{45_8} = 17\mathrm{s}^{-1}$.

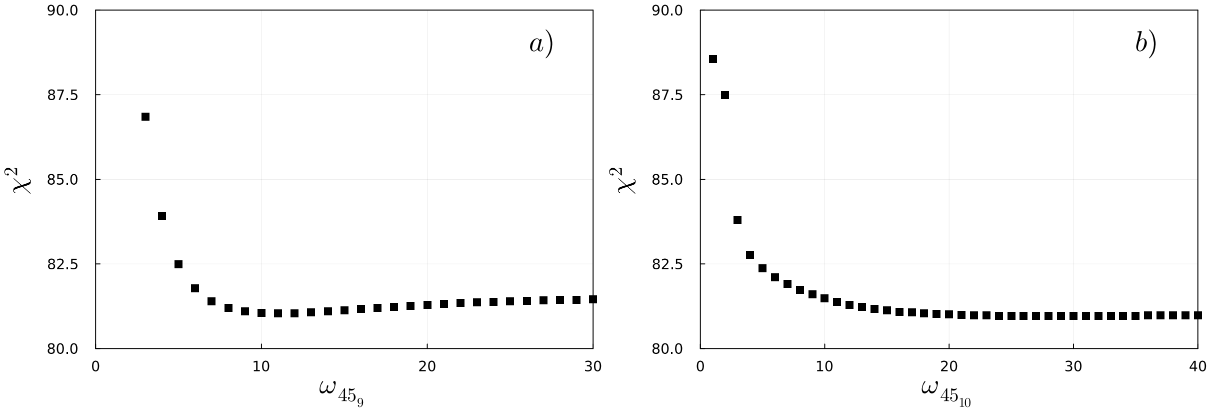

Figure P: **Determination of kinetic rates ω_{45_9} and $\omega_{45_{10}}$ from fluorescence signatures of mRNAs that consist of 26 codons for random IFI input vectors.** It is assumed that all kinetic rates are known except for the rate $\omega_{45_{9(10)}}$ for codon 9 (10). Plot of the goodness-of-fit parameter χ^2 versus the unknown kinetic rate a): ω_{45_9} and b): $\omega_{45_{10}}$. The local minima are found at $\omega_{45_9} = 11\text{s}^{-1}$ and $\omega_{45_{10}} = 28\text{s}^{-1}$. The input rates used to simulate the fluorescence signatures are $\omega_{45_9} = 15\text{s}^{-1}$ and $\omega_{45_{10}} = 15\text{s}^{-1}$.

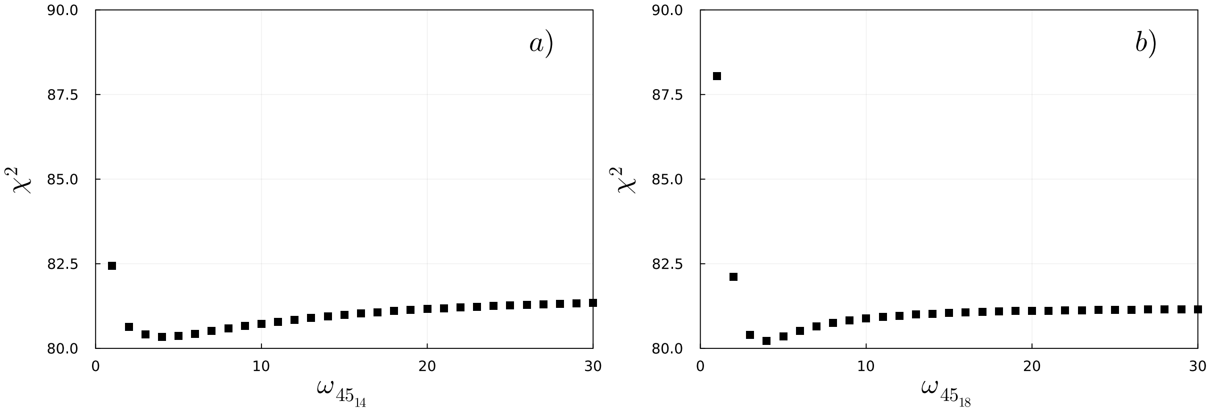

Figure Q: **Determination of kinetic rates $\omega_{45_{14}}$ and $\omega_{45_{18}}$ from fluorescence signatures of mRNAs that consist of 26 codons for random IFI input vectors.** It is assumed that all kinetic rates are known except for the rate $\omega_{45_{14(18)}}$ for codon 14 (18). Plot of the goodness-of-fit parameter χ^2 versus the unknown kinetic rate a): $\omega_{45_{14}}$ and b): $\omega_{45_{18}}$. The local minima are found at $\omega_{45_{14}} = 4\text{s}^{-1}$ and $\omega_{45_{18}} = 4\text{s}^{-1}$. The input rates used to simulate the fluorescence signatures are $\omega_{45_{14}} = 19\text{s}^{-1}$ and $\omega_{45_{18}} = 24\text{s}^{-1}$.

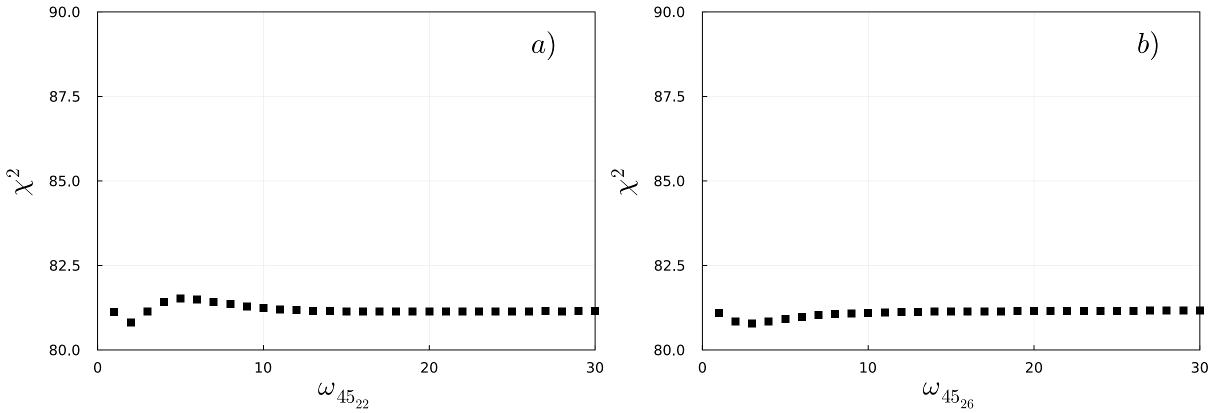

Figure R: **Determination of kinetic rates $\omega_{45_{22}}$ and $\omega_{45_{26}}$ from fluorescence signatures of mRNAs that consist of 26 codons for random IFI input vectors.** It is assumed that all kinetic rates are known except for the rate $\omega_{45_{22(26)}}$ for codon 22 (26). Plot of the goodness-of-fit parameter χ^2 versus the unknown kinetic rate a): $\omega_{45_{22}}$ and b): $\omega_{45_{26}}$. The local minima are found at $\omega_{45_{22}} = 2\text{s}^{-1}$ and $\omega_{45_{26}} = 3\text{s}^{-1}$. The input rates used to simulate the fluorescence signatures are $\omega_{45_{22}} = 18\text{s}^{-1}$ and $\omega_{45_{26}} = 14\text{s}^{-1}$.

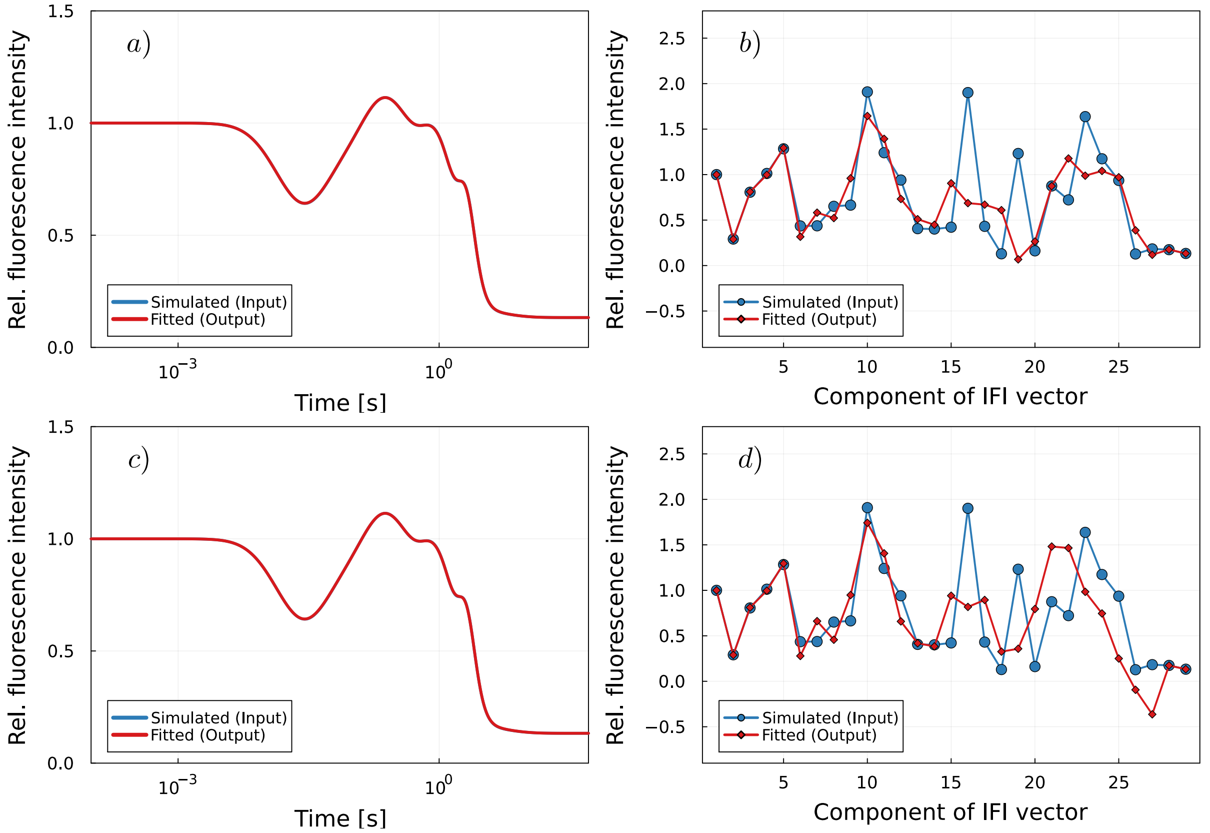

Figure S: **Fluorescence signatures of mRNAs that consist of 26 codons for random IFI input vectors.** a, c): The simulated fluorescence signature is compared to the best theoretical fit in terms of least squares. The theoretical model and simulated data curves are in perfect agreement. b, d): Fitted IFIs obtained from the analysis of the fluorescence signature compared to the given IFI input vector. The rates ω_{45_1} (a, b) and $\omega_{45_{18}}$ (c, d) were treated as unknown fitting parameters.

References

[1] P. Bevington and D.K. Robinson. *Data Reduction and Error Analysis for the Physical Sciences*, volume 3 (145-147). McGraw-Hill Education, 2003.

[2] H.M. Taylor and S. Karlin. *An introduction to stochastic modeling.* Academic Press, 1985.

[3] S. Rudorf, M. Thommen, M.V. Rodnina, and R. Lipowsky. Deducing the kinetics of protein synthesis in vivo from the transition rates measured in vitro. *PLoS Computational Biology*, 10(10):e1003909, 10 2014. doi: 10.1371/journal.pcbi.1003909.
