# Supplementary material for: Decomposing bulk signals to reveal hidden information in processive enzyme reactions: A case study in mRNA translation
Source: PLoS Comput Biol. 2024 Mar 5;20(3):e1011918. doi: 10.1371/journal.pcbi.1011918 (PMC10942256; doi:10.1371/journal.pcbi.1011918)
Supplement: S2 Text — (PDF) [file pcbi.1011918.s002.pdf]

Supplemental Information

Decomposing bulk signals to reveal hidden information in processive enzyme reactions: A case study in mRNA translation

Nadin Haase[1], Wolf Holtkamp[2,3], Simon Christ[1], Dag Heinemann[4,5,6], Marina V. Rodnina[2], and Sophia Rudorf[1,*]

[1]Leibniz University Hannover, Institute of Cell Biology and Biophysics, Germany
[2]Max Planck Institute for Multidisciplinary Sciences, Department of Physical Biochemistry, Germany
[3]Paul-Ehrlich-Institut, Division of Allergology, Germany.
[4]Leibniz University Hannover, Hannover Centre for Optical Technologies (HOT), Germany
[5]Leibniz University Hannover, Institute of Horticultural Production Systems, Germany
[6]Leibniz University Hannover, PhoenixD Cluster of Excellence, Germany
[*]rudorf@cell.uni-hannover.de

Decomposition of fluorescence signatures of mRNAs with different lengths

In this paragraph, we analyze simulated ensemble fluorescence signatures corresponding to translation of mRNAs consisting of 8, 12, 16 and 20 identical codons as well as codons with non-uniform translation rates (see Fig. A - Fig. H). The rate ω_{45_x} is set to $\omega_{45} = 13.8\,\mathrm{s}^{-1}$ for all codons in case of uniform translation. For non-uniform translation, the rates ω_{45_x} are codon dependent as shown in Table A.

Table A: For each codon, a different rate ω_{45_x} is used in the Markov model description of the translation process, resulting in non-uniform translation elongation rates.

Rate	Value
ω_{45_1}	80 s^{-1}
ω_{45_2}	11 s^{-1}
ω_{45_3}	43 s^{-1}
ω_{45_4}	2 s^{-1}
ω_{45_5}	13 s^{-1}
ω_{45_6}	15 s^{-1}
ω_{45_7}	10 s^{-1}
ω_{45_8}	17 s^{-1}
ω_{45_9}	15 s^{-1}
$\omega_{45_{10}}$	15 s^{-1}
$\omega_{45_{11}}$	25 s^{-1}
$\omega_{45_{12}}$	35 s^{-1}
$\omega_{45_{13}}$	49 s^{-1}
$\omega_{45_{14}}$	19 s^{-1}
$\omega_{45_{15}}$	33 s^{-1}
$\omega_{45_{16}}$	28 s^{-1}
$\omega_{45_{17}}$	7 s^{-1}
$\omega_{45_{18}}$	24 s^{-1}
$\omega_{45_{19}}$	10 s^{-1}
$\omega_{45_{20}}$	10 s^{-1}

8-codon-mRNA

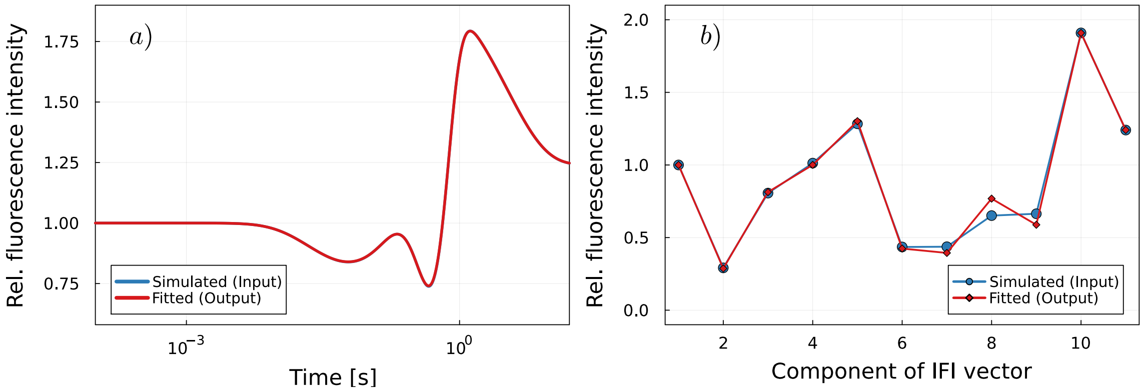

Figure A: **Fluorescence signature of an mRNA that consists of 8 codons for random IFI input vector and uniform translation.** a): The simulated fluorescence signature is compared to the best theoretical fit in terms of least squares. The theoretical model and simulated data curves are in perfect agreement. b): Fitted IFIs obtained from the analysis of the fluorescence signature compared to the given IFI input vector. The rate ω_{45} is set to $\omega_{45} = 13.8\mathrm{s}^{-1}$ for all codons. Regularization is applied with regularization parameter $\alpha = 0.0326$.

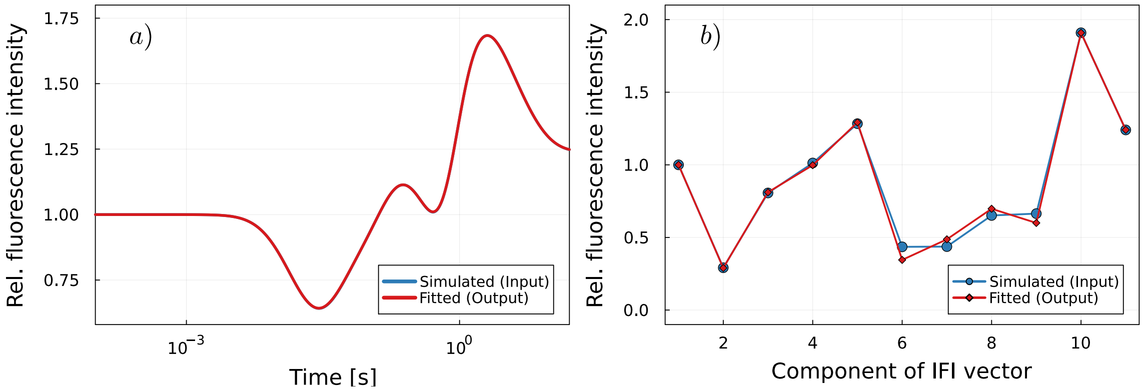

Figure B: **Fluorescence signature of an mRNA that consists of 8 codons for random IFI input vector and non-uniform translation.** a): The simulated fluorescence signature is compared to the best theoretical fit in terms of least squares. The theoretical model and simulated data curves are in perfect agreement. b): Fitted IFIs obtained from the analysis of the fluorescence signature compared to the given IFI input vector. The rate ω_{45} is different for each codon, see Table A. Regularization is applied with regularization parameter $\alpha = 0.0188$.

12-codon-mRNA

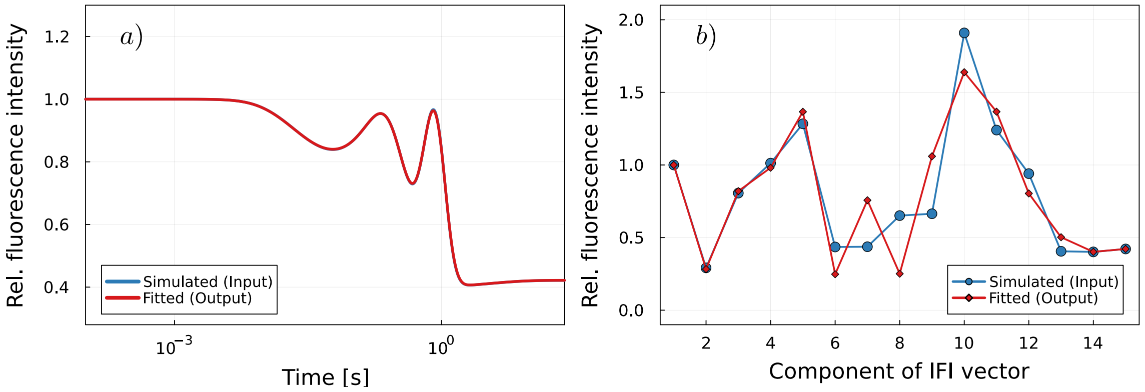

Figure C: **Fluorescence signature of an mRNA that consists of 12 codons for random IFI input vector and uniform translation.** a): The simulated fluorescence signature is compared to the best theoretical fit in terms of least squares. The theoretical model and simulated data curves are in perfect agreement. b): Fitted IFIs obtained from the analysis of the fluorescence signature compared to the given IFI input vector. The rate ω_{45} is set to $\omega_{45} = 13.8\text{s}^{-1}$ for all codons. Regularization is applied with regularization parameter $\alpha = 0.0103$.

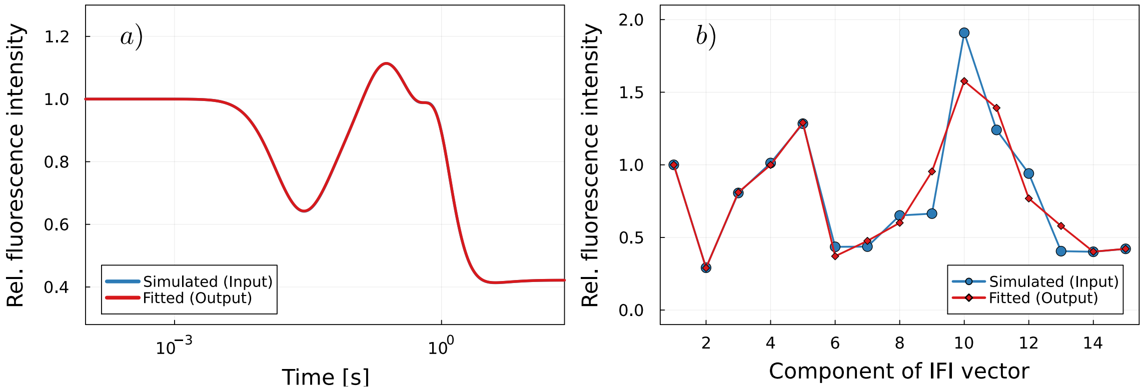

Figure D: **Fluorescence signature of an mRNA that consists of 12 codons for random IFI input vector and non-uniform translation.** a): The simulated fluorescence signature is compared to the best theoretical fit in terms of least squares. The theoretical model and simulated data curves are in perfect agreement. b): Fitted IFIs obtained from the analysis of the fluorescence signature compared to the given IFI input vector. The rate ω_{45} is different for each codon, see Table A. Regularization is applied with regularization parameter $\alpha = 0.0148$.

16-codon-mRNA

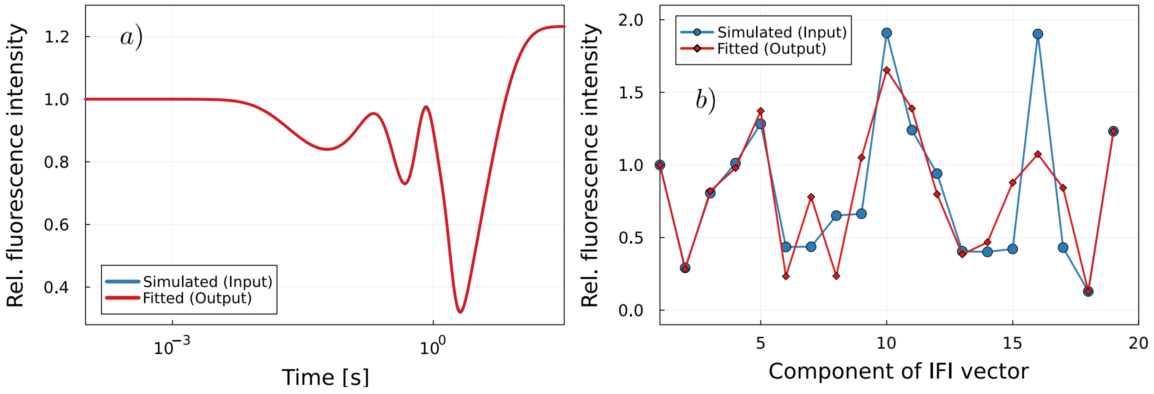

Figure E: **Fluorescence signature of an mRNA that consists of 16 codons for random IFI input vector and uniform translation.** a): The simulated fluorescence signature is compared to the best theoretical fit in terms of least squares. The theoretical model and simulated data curves are in perfect agreement. b): Fitted IFIs obtained from the analysis of the fluorescence signature compared to the given IFI input vector. The rate ω_{45} is set to $\omega_{45} = 13.8\mathrm{s}^{-1}$ for all codons. Regularization is applied with regularization parameter $\alpha = 0.0096$.

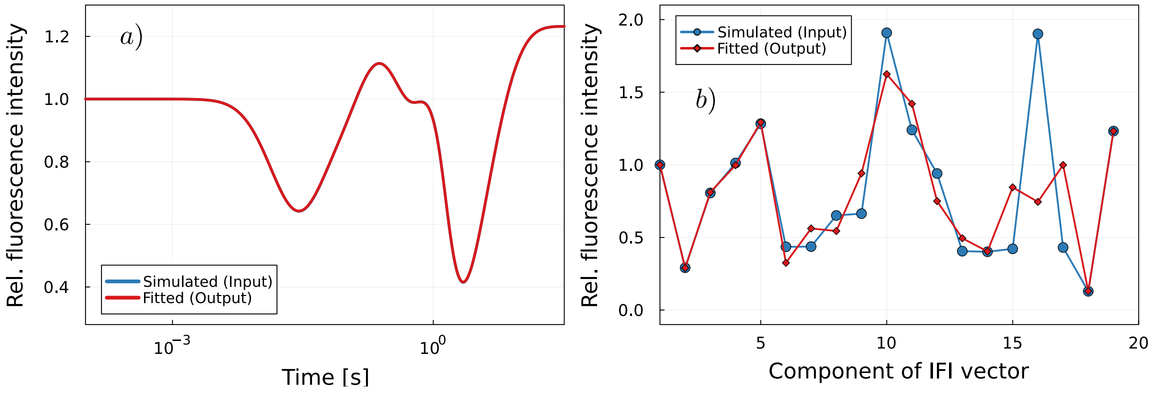

Figure F: **Fluorescence signature of an mRNA that consists of 16 codons for random IFI input vector and non-uniform translation.** a): The simulated fluorescence signature is compared to the best theoretical fit in terms of least squares. The theoretical model and simulated data curves are in perfect agreement. b): Fitted IFIs obtained from the analysis of the fluorescence signature compared to the given IFI input vector. The rate ω_{45} is different for each codon, see Table A. Regularization is applied with regularization parameter $\alpha = 0.0099$.

20-codon-mRNA

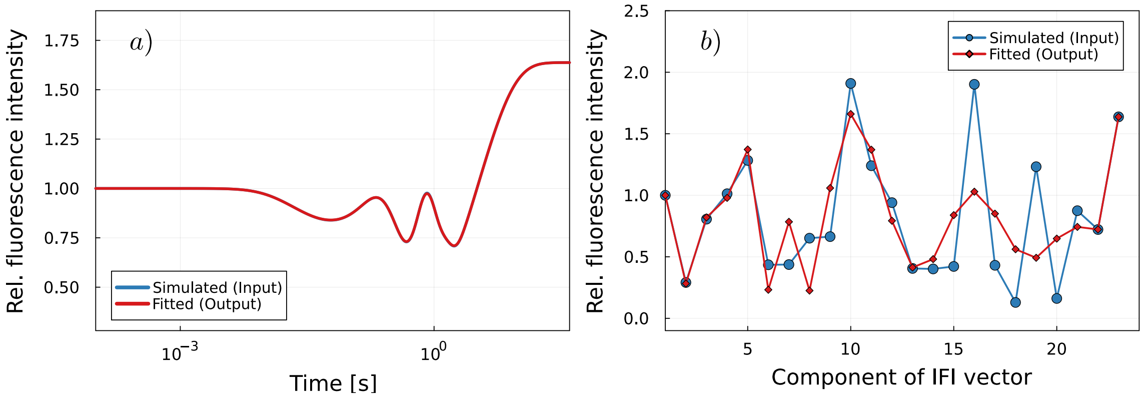

Figure G: **Fluorescence signature of an mRNA that consists of 20 codons for random IFI input vector and uniform translation.** a): The simulated fluorescence signature is compared to the best theoretical fit in terms of least squares. The theoretical model and simulated data curves are in perfect agreement. b): Fitted IFIs obtained from the analysis of the fluorescence signature compared to the given IFI input vector. The rate ω_{45} is set to $\omega_{45} = 13.8\text{s}^{-1}$ for all codons. Regularization is applied with regularization parameter $\alpha = 0.0096$.

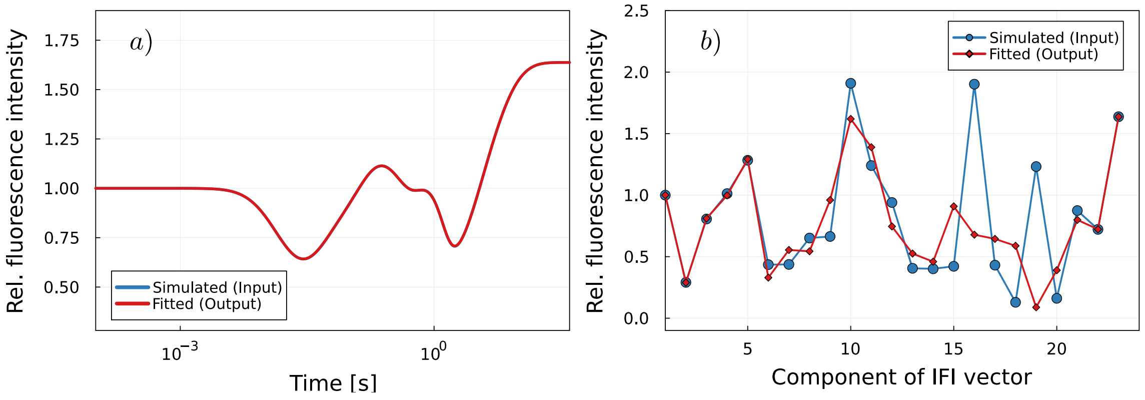

Figure H: **Fluorescence signature of an mRNA that consists of 20 codons for random IFI input vector and non-uniform translation.** a): The simulated fluorescence signature is compared to the best theoretical fit in terms of least squares. The theoretical model and simulated data curves are in perfect agreement. b): Fitted IFIs obtained from the analysis of the fluorescence signature compared to the given IFI input vector. The rate ω_{45} is different for each codon, see Table A. Regularization is applied with regularization parameter $\alpha = 0.0099$.

LepB mRNA

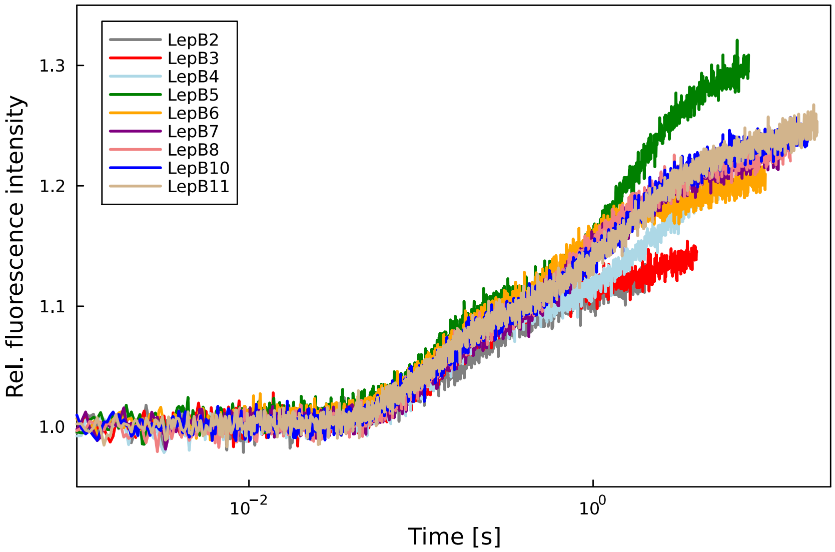

Figure I: **Fluorescence signatures of LepB mRNAs.** Measured fluorescence signatures of the *in-vitro* translation of LepB mRNAs containing the first 2 - 11 codons. (Data for LepB9 mRNA translation were not available.)

Table B: Apart from the rates ω_{45}, all transition rates ω_{ij} are used as presented in Table 2 in the main paper. The codon-specific rate of translation ω_{elo} for each codon is calculated by first step analysis [1]. For each codon x, the rate ω_{45_x} is fitted during the computational analysis (see *Determination of kinetic rate* ω_{45} for more information). It is assumed that all kinetic rates are known except for the fitted rate. For short mRNAs, the fitting of kinetic rates and IFI values at the same time yields a unique solution, while the analysis gets more ambiguous for longer mRNAs (see Fig. L and M). *: No data curve was available for LepB9. The rate ω_{45_8} is therefore not directly fitted from a data curve but concluded from reasonable IFI values for the subsequent analysis.

mRNA	Rate	Fitted value ω_{45} [s^{-1}]	Codon specific ω_{elo} from input ω_{45} [s^{-1}]
LepB2	ω_{45_1}	5.2 ± 0.3	4.5 ± 0.3
LepB3	ω_{45_2}	3.6 ± 0.8	3.3 ± 0.7
LepB4	ω_{45_3}	2.2 ± 0.4	2.1 ± 0.4
LepB5	ω_{45_4}	0.8 ± 0.3	0.8 ± 0.3
LepB6	ω_{45_5}	5.1 ± 0.5	4.5 ± 0.4
LepB7	ω_{45_6}	0.9 (- 0.5 + 0.9)	0.9 (- 0.5 + 0.8)
LepB8	ω_{45_7}	0.6 (- 0.5 + 1.8)	0.6 (- 0.5 + 1.6)
LepB9	ω_{45_8}	1*	1
LepB10	ω_{45_9}	1.2 ± 0.6	1.2 ± 0.6
LepB11	$\omega_{45_{10}}$	0.2 (- 0.1 + 0.9)	0.2 (- 0.1 + 0.9)

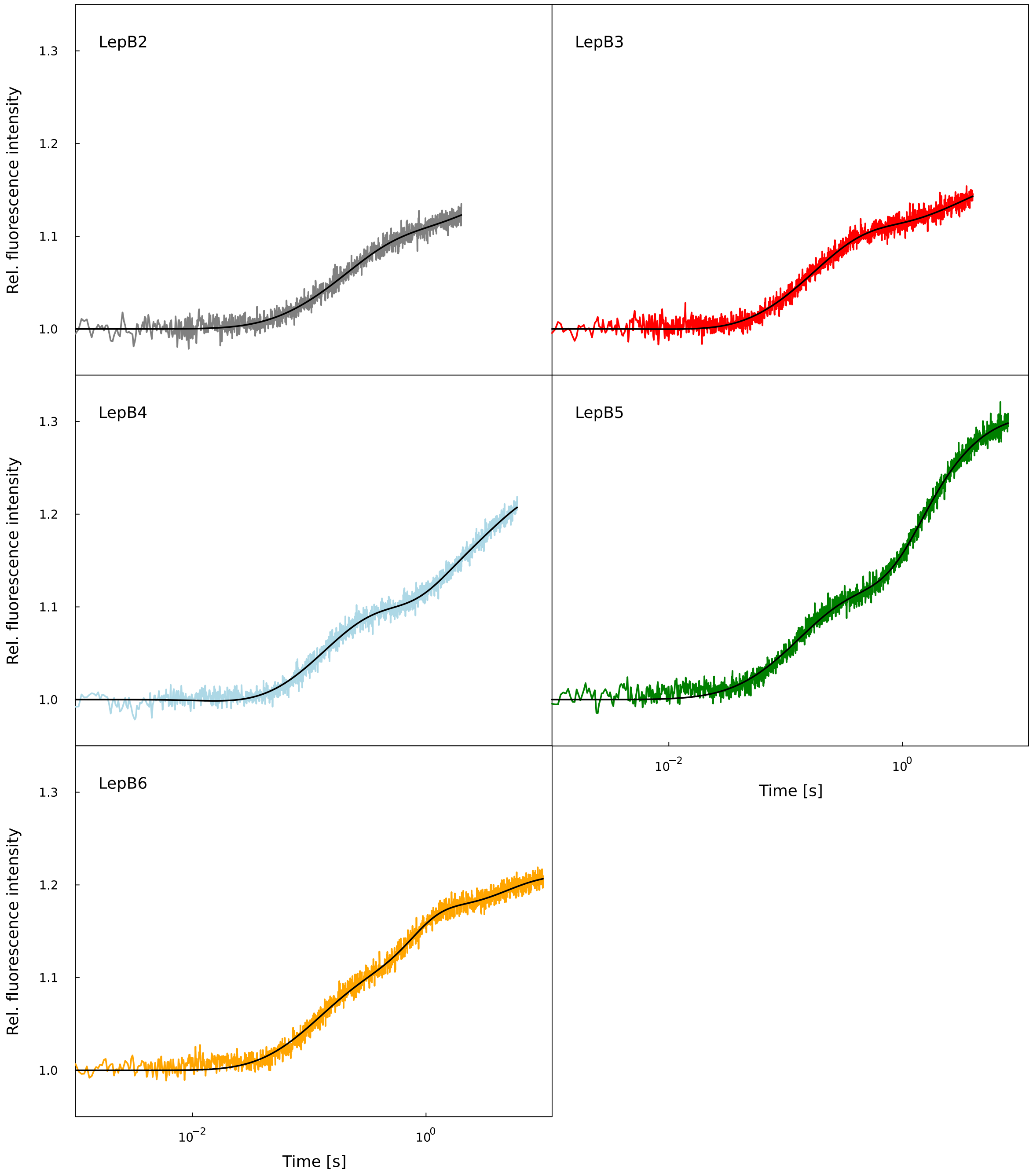

Figure J: **Decomposition of fluorescence signatures of LepB mRNAs.** Measured fluorescence signatures of the *in-vitro* translation of LepB mRNA (colored lines) and best fit in terms of least squares (black lines) for LepB2-LepB6.

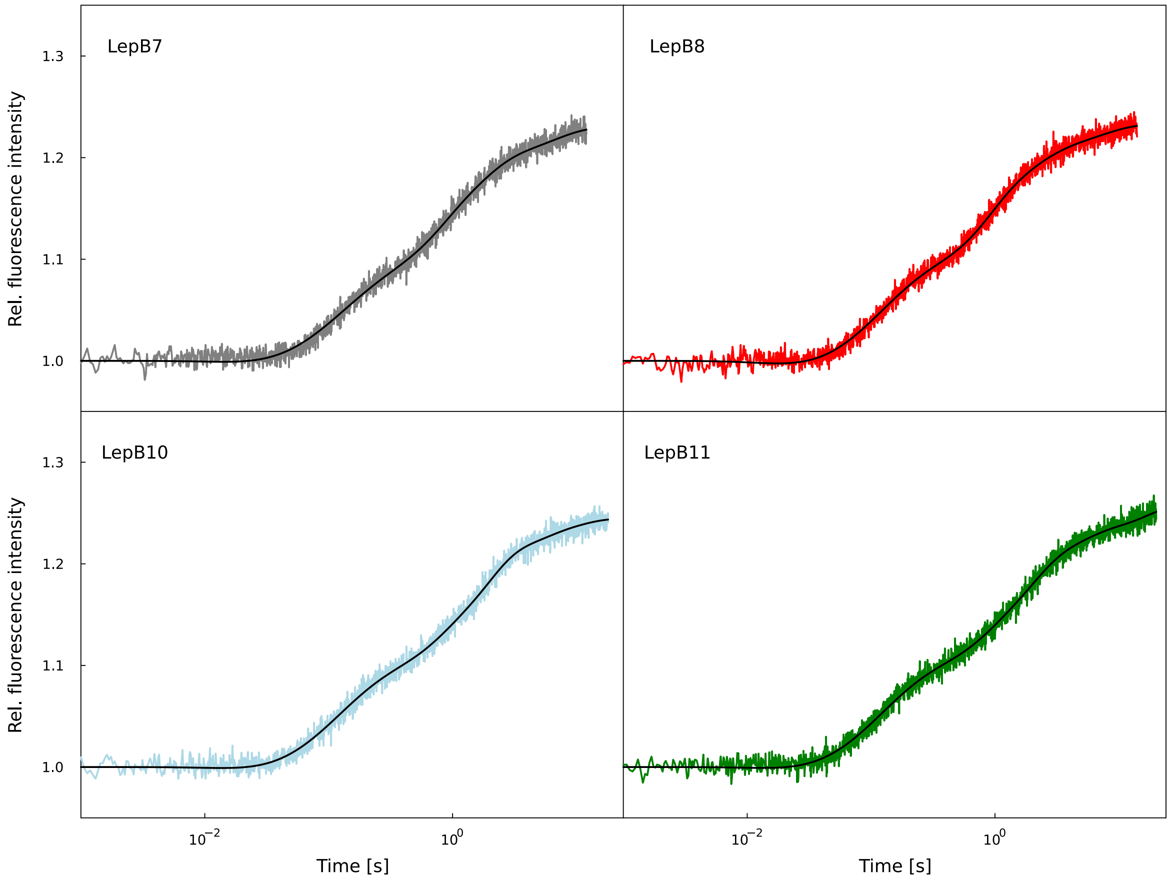

Figure K: **Decomposition of fluorescence signatures of LepB mRNAs.** Measured fluorescence signatures of the *in-vitro* translation of LepB mRNA (colored lines) and best fit in terms of least squares (black lines) for LepB7, LepB8, LepB10 and LepB11.

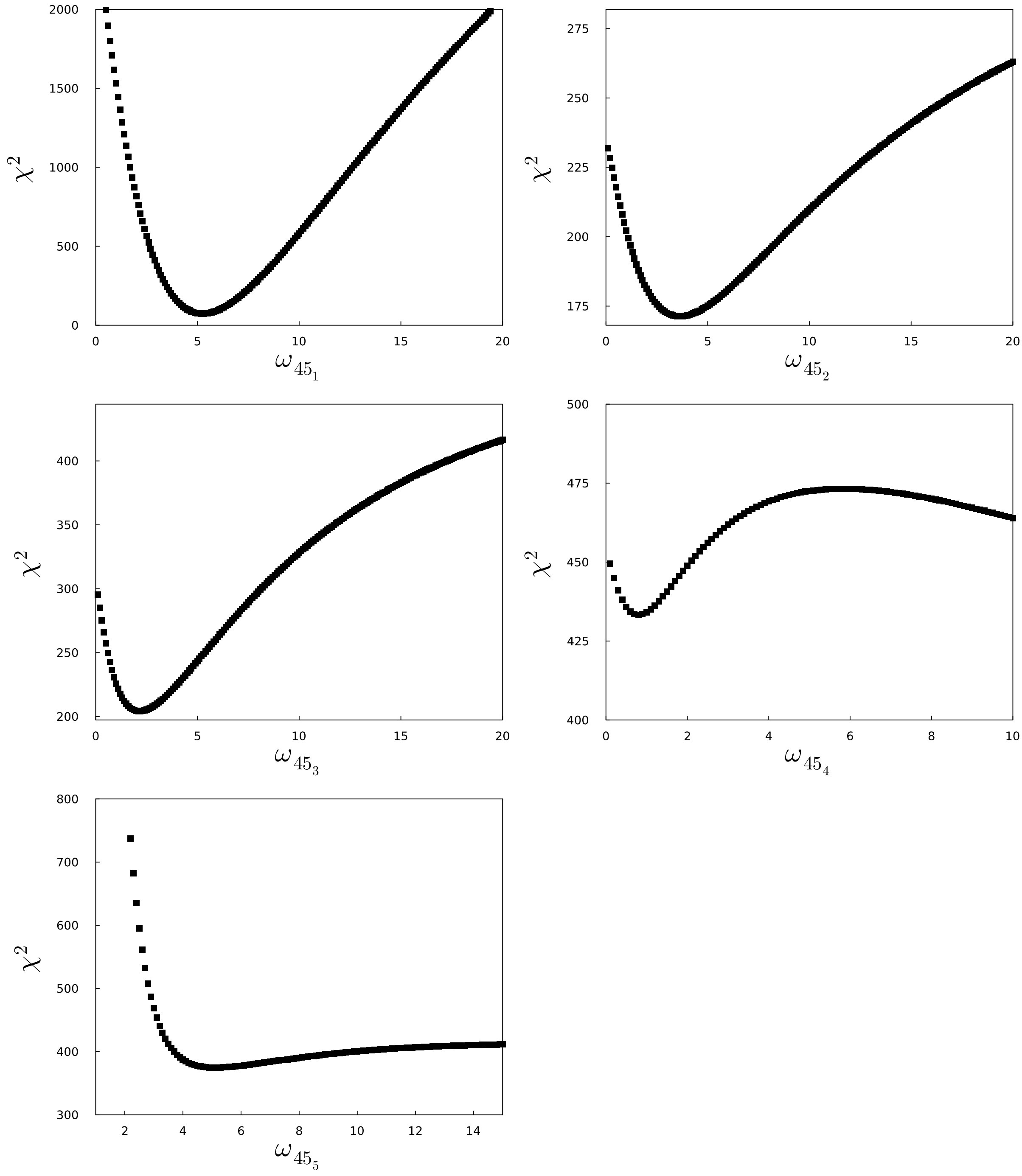

Figure L: **Determination of kinetic rates ω_{45_x} from fluorescence signatures of LepB2 to LepB6 translation.** It is assumed that all kinetic rates are known except for the rate ω_{45_x} for codon x. Plot of the goodness-of-fit parameter χ^2 versus the unknown kinetic rate.

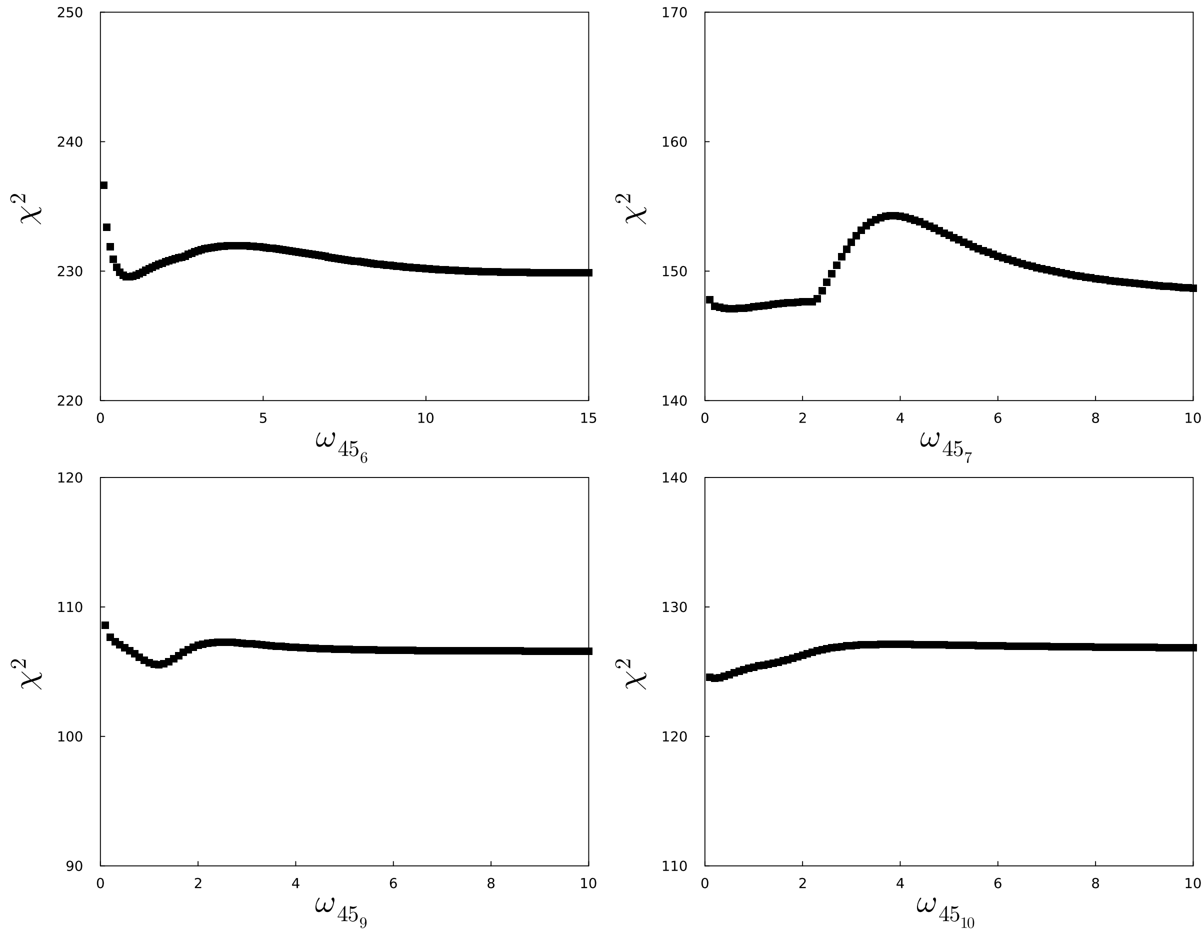

Figure M: **Determination of kinetic rates ω_{45_x} from fluorescence signatures of LepB7, LepB8, LepB10 and LepB11 translation.** It is assumed that all kinetic rates are known except for the rate ω_{45_x} for codon x. Plot of the goodness-of-fit parameter χ^2 versus the unknown kinetic rate.

Singular Value Decomposition and condition number

For a rectangular matrix $\mathbf{A} \in \mathbb{R}^{m \times n}$ with $m \geq n$, the singular value decomposition (SVD) of $\mathbf{A}$ is of the form [2, 3]

$$\mathbf{A} = \mathbf{U}\boldsymbol{\Sigma}\mathbf{V}^{\mathrm{T}} = \sum_{i=1}^{n} \mathbf{u}_i \sigma_i \mathbf{v}_i^{\mathrm{T}} \tag{1}$$

where $\mathbf{U} = (\mathbf{u}_1, \ldots, \mathbf{u}_n)$ is an $m \times n$ matrix and $\mathbf{V} = (\mathbf{v}_1, \ldots, \mathbf{v}_n)$ is an $n \times n$ matrix with orthonormal columns, so that $\mathbf{U}^{\mathrm{T}}\mathbf{U} = \mathbf{V}^{\mathrm{T}}\mathbf{V} = \mathbf{I_n}$. $\boldsymbol{\Sigma}$ is an $n \times n$ diagonal matrix with the non-negative singular values of $\mathbf{A}$ of the ordering $\sigma_1 \geq \ldots \geq \sigma_n \geq 0$. The 2-norm *condition number* of $\mathbf{A}$ is defined as [3, 4]

$$\kappa(\mathbf{A}) = ||\mathbf{A}||_2 ||\mathbf{A}^{-1}||_2 = \frac{\sigma_1(\mathbf{A})}{\sigma_n(\mathbf{A})}\,. \tag{2}$$

Tikhonov Regularization

Consider a system of linear equations $\mathbf{Ax} = \mathbf{b}$ with $\mathbf{A} \in \mathbb{R}^{m \times n}$, $\mathbf{x} \in \mathbb{R}^n$, $\mathbf{b} \in \mathbb{R}^m$, and $m > n$. The vector $\mathbf{x}$ is unknown. Such a problem is called ill-posed if [3, 5]

1. the singular values of $\mathbf{A}$ decay gradually to zero (see Eq. 1), and
2. the condition number of $\mathbf{A}$ is large (see Eq. 2).

The purpose of regularization is to introduce additional information in order to solve an ill-posed problem. In the Tikhonov regularization method a damping is added to filter out the components corresponding to the small singular values [5]. The standard-form version of Tikhonov's method takes the form

$$\mathbf{x}_\alpha = \operatorname{argmin}\{||\mathbf{Ax} - \mathbf{b}||_2^2 + \alpha^2 ||\mathbf{x}||_2^2\}, \tag{3}$$

where α is a positive constant called the regularization parameter. To filter out the contributions corresponding to the small singular values the filter factor f_i is included in the solution [3]

$$\mathbf{x}_\alpha = \sum_{i=1}^{n} f_i \frac{\mathbf{u}_i^{\mathrm{T}}\mathbf{b}}{\sigma_i} \mathbf{v}_i. \tag{4}$$

For Tikhonov regularization, the filter factor is determined by the singular values and the regularization parameter [3]

$$f_i = \frac{\sigma_i^2}{(\sigma_i^2 + \alpha^2)}. \tag{5}$$

References

[1] S. Rudorf, M. Thommen, M.V. Rodnina, and R. Lipowsky. Deducing the kinetics of protein synthesis in vivo from the transition rates measured in vitro. *PLoS Computational Biology*, 10(10):e1003909, 10 2014. doi: 10.1371/journal.pcbi.1003909.

[2] G. Strang. *Introduction To Linear Algebra.* Wellesley-Cambridge Press, third edition, 2003. Chapter 6.

[3] C. Hansen. *Rank-Deficient and Discrete Ill-Posed Problems: Numerical Aspects of Linear Inversion (Monographs on Mathematical Modeling and Computation).* Society for Industrial and Applied Mathematics, 1998.

[4] W. Cheney and D. Kincaid. *Numerical Mathematics and Computing.* Brooks/Cole CENGAGE Learning, 7th edition, 2004.

[5] G.H. Golub, C. Hansen, and D.P. O'Leary. Tikhonov Regularization And Total Least Squares. *SIAM Journal on Matrix Analysis and Applications*, 21(1):185–194, 1999. doi: 10.1137/S0895479897326432.
